# Exosomal CCT6A Secreted by Cancer‐Associated Fibroblasts Interacts with β‐Catenin to Enhance Chemoresistance and Tumorigenesis in Gastric Cancer

**DOI:** 10.1002/advs.202506674

**Published:** 2025-08-13

**Authors:** Hui Sun, Tianqi Zhang, Xiaoyan Zhang, Yingxue Liu, Xu Wang, Xin Wang, Cong Tan, Shujuan Ni, Weiwei Weng, Meng Zhang, Lei Wang, Dan Huang, Xiaoyu Wang, Wenfeng Wang, Weiqi Sheng, Mi‐die Xu

**Affiliations:** ^1^ Department of Pathology Fudan University Shanghai Cancer Center Shanghai 200032 China; ^2^ Department of Oncology Shanghai Medical College Fudan University Shanghai 200032 China; ^3^ Institute of Pathology Fudan University Shanghai 200032 China; ^4^ Experiment Center for Science and Technology Shanghai University of Traditional Chinese Medicine Shanghai 201203 China; ^5^ Shanghai Urological Cancer Institute Cancer Institute Fudan University Shanghai Cancer Center Fudan University Shanghai 200032 China

**Keywords:** cancer‐associated fibroblast, CCT6A, chemoresistance, c‐Myc, gastric cancer

## Abstract

Cancer‐associated fibroblasts (CAFs) are pivotal components of the tumor microenvironment that drive gastric cancer (GC) progression and chemoresistance. Here, CCT6A is identified as a CAF‐derived factor whose high expression is positively associated with GC progression and plays a key regulatory role in stemness, chemoresistance, and glucose metabolism. In a co‐culture system of CAFs and cancer cells, it is discovered that CCT6A is transferred from CAFs to tumor cells mainly via exosomal transport, thereby enhancing stemness, chemoresistance, and glycolysis. Mechanistically, CCT6A interacts with β‐catenin, inducing its phosphorylation and nuclear translocation, which subsequently leads to transcriptional suppression of the glycolysis inhibitors DDIT4 and TXNIP through c‐Myc activation. Furthermore, a feedforward regulatory loop is uncovered in which the CCT6A pseudogene CCT6P1 acts as a competitive endogenous RNA (ceRNA) by sequestering miR‐922, thereby stabilizing CCT6A expression, while c‐Myc co‐activates both CCT6A and CCT6P1, amplifying oncogenic signaling. Collectively, the findings not only reveal the pivotal mediator of CAF‐secreted CCT6A in orchestrating stemness, chemoresistance, and metabolic reprogramming in GC but also highlight its dual utility as a diagnostic biomarker and therapeutic target.

## Graphical abstract

Here, it is revealed that CAF‐secreted exosomal CCT6A enhances stemness, chemoresistance, and glycolysis in GC via the β‐catenin/c‐Myc/DDIT4‐TXNIP axis. Furthermore, CCT6P1, a pseudogene of CCT6A, is identified as upregulating CCT6A by competitively binding miR‐922, while c‐Myc transcriptionally activates both CCT6P1 and CCT6A. Collectively, the findings highlight CCT6A as a promising therapeutic target for CAF‐directed interventions in GC.

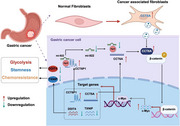

## 1 Introduction

Gastric cancer (GC) ranks as the fifth most common malignancy and the second leading cause of global cancer‐related mortality, accounting for over 1 million new cases and ≈770 000 deaths annually.^[^ ^1^ ^]^ China bears a particularly high burden of GC incidence and mortality.^[^ ^2^ ^]^ While multimodual therapies have advanced, chemotherapy remains the mainstay for locally advanced and metastatic GC. However, the development of chemoresistance severely limits treatment efficacy,^[^ ^3^ ^]^ highlighting the urgent need to: 1) decipher resistance mechanisms, 2) identify predictive biomarkers, and 3) develop strategies to overcome treatment resistance. Addressing these challenges could enable early detection of resistant tumors, guide timely interventions to prevent recurrence, and reveal novel therapeutic targets to improve patient outcomes.

Cancer‐associated fibroblasts (CAFs), a predominant component of the tumor stroma in GC, originate from normal fibroblasts (NFs) that become activated in response to tumor‐derived signals.^[^ ^4^ ^]^ CAFs not only constitute a major portion of the tumor microenvironment (TME) but also orchestrate complex molecular interactions that drive GC malignancy.^[^ ^5^ ^]^ Emerging evidence indicates that CAFs play an important role in mediating drug resistance in GC through various mechanisms, including extracellular matrix remodeling, secretion of pro‐tumorigenic factors that enhance angiogenesis and epithelial‐mesenchymal transition (EMT), metabolic reprogramming to support tumor proliferation and metastasis, and induction of an immunosuppressive microenvironment.^[^ ^5^, ^6^, ^7^ ^]^ For example, CAFs activate YAP signaling in GC cells to promote 5‐Fu chemoresistance.^[^ ^8^ ^]^ Concurrently, our previous work demonstrated that CAFs secrete IL‐8 to promote oxaliplatin resistance in GC cells via activation of the CXCR2/PI3K/Akt pathway, and that PDPN(+) CAFs induce angiogenesis in GC via AKT/NF‐κB activation and the CCL2‐ACKR1 axis.^[^ ^9^, ^10^ ^]^

The chaperonin‐containing TCP1 complex (CCT) is a molecular chaperone family that plays a pivotal role in regulating cell cycle progression, growth, and migration of cancer cells. CCT6A, the ζ‐subunit of the CCT complex, is a secreted protein composed of two stacked identical rings.^[^ ^11^ ^]^ Its expression is frequently upregulated in various malignancies, including non‐small cell lung cancer (NSCLC), glioma, melanoma, colorectal cancer (CRC), and testicular cancer, largely due to amplification of the 7p11.2 chromosomal region.^[^ ^12^, ^13^, ^14^ ^]^ Functionally, CCT6A has been shown to promote NSCLC progression by inhibiting SMAD2 and activating pro‐metastatic TGF‐β signaling,^[^ ^15^ ^]^ while exosomal CCT6A serves as a potential prognostic biomarker in glioblastoma.^[^ ^16^ ^]^ Additionally, CCT6A functions as an oncogene in lung cancer^[^ ^17^ ^]^ and is implicated in T cell exhaustion within the TME of CRC.^[^ ^18^ ^]^ Previous studies have highlighted significant overexpression of CCT6A in epithelial tissue, correlating with poor prognosis in GC.^[^ ^19^ ^]^ However, the specific role and underlying mechanism of CCT6A in GC remain largely unknown, warranting further investigation.

Here, we identify CCT6A as a key CAF‐derived factor strongly associated with chemoresistance, metabolic reprogramming, and poor prognosis in GC. Through functional and mechanistic analyses, we demonstrate that CAF‐derived exosomal CCT6A promotes GC cell stemness, cisplatin resistance, and glycolysis via the β‐catenin/DDIT4‐TXNIP/c‐Myc axis. Notably, we uncover a novel competing endogenous RNA (ceRNA) mechanism in which the CCT6A pseudogene, CCT6P1, sponges miR‐922 to upregulate CCT6A. Furthermore, c‐Myc transcriptionally co‐activates both CCT6P1 and CCT6A, creating a self‐reinforcing oncogenic loop. Collectively, our findings reveal a CAF‐driven axis of chemoresistance and metabolic adaptation in GC, nominating CCT6A as a promising therapeutic target for stromal‐mediated tumor progression.

## 2 Result

### 2.1 Expression of CCT6A in CAFs Correlates with Chemoresistance, Glucose Metabolism, and Poor Survival in GC

**Figure 1:**
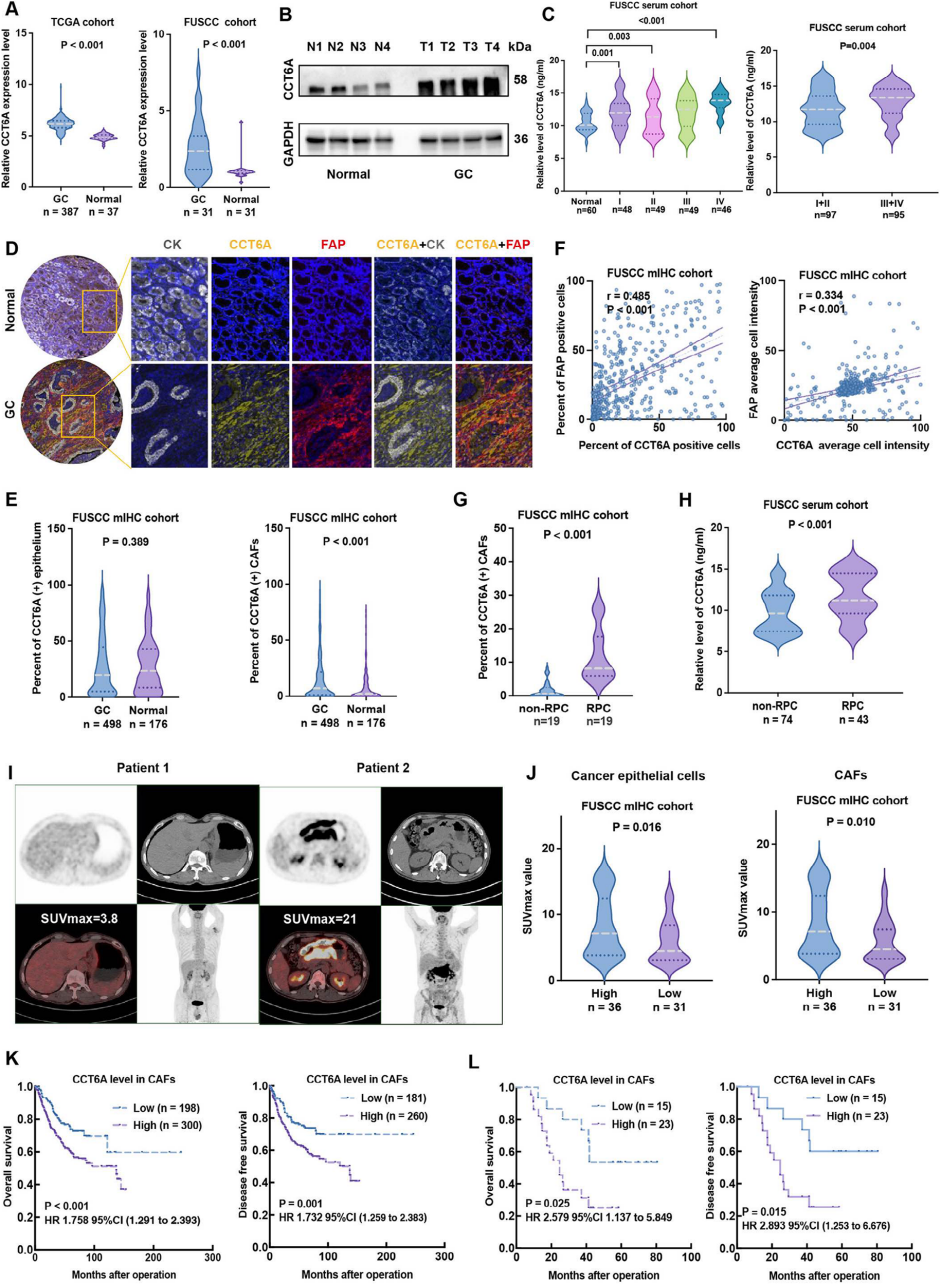
CCT6A expression in CAFs correlates with chemoresistance, glucose metabolism, and poor survival in gastric cancer patients. A) Box plots comparing CCT6A mRNA levels in gastric cancer (GC) tissues versus normal tissues from TCGA and FUSCC cohorts. B) Western blot analysis of CCT6A protein expression in four paired GC (T) and adjacent normal tissues (N) from the same patient. C) ELISA‐based quantification of serum CCT6A levels in GC patients versus healthy controls. D‐E) Representative multiplexed immunohistochemistry (mIHC) staining (D) and quantitative analysis (E) of CCT6A expression in GC and adjacent normal tissues. F) Pearson correlation analysis between the percentage and average fluorescence intensity of CCT6A and FAP expression in GC tissues. G,H) mIHC staining (G) and ELISA H) demonstrating CCT6A levels in RPC group versus non‐RPC group. I,J) PET/CT imaging I) and quantitative analysis J) showing higher SUVmax values in cancer epithelial cells and CAFs from patients with high versus low CCT6A immunostaining. K,L) Kaplan‐Meier analysis of overall survival (OS) and disease‐free survival (DFS) in GC patients stratified by CAF CCT6A expression (mIHC) after surgical resection (K) or surgery plus adjuvant therapy (L). (Data are mean±SEM. *p*‐values calculated using Mann–Whitney U‐test, one‐way ANOVA, Pearson's r, or log‐rank test).

Analysis of TCGA GC dataset and RT‐qPCR from the FUSCC cohort revealed significantly elevated CCT6A expression in gastric adenocarcinoma compared to normal gastric tissues (*p* < 0.001, **Figure** **1**A). This finding was further validated by immunoblot analysis (Figure 1B). Given that CCT6A is a secreted protein, we also quantified its serum level in human volunteers. ELISA demonstrated markedly higher circulating CCT6A in GC patients compared to healthy controls (*p* = 0.001, *p* = 0.003, and *p* < 0.001, Figure 1C). Notably, advanced‐stage GC patients (TNM stage III + IV) displayed significantly elevated serum CCT6A levels compared to those with early‐stage cases (*p* = 0.004, Figure 1C). These findings position CCT6A as a promising diagnostic and prognostic biomarker for GC.

Dual immunohistochemical (IHC) analysis indicated that CCT6A is localized in the cancer epithelium and stromal compartments (Figure S1A, Supporting Information). Given previous findings identifying fibroblast activation protein (FAP) as a CAF marker in GC,^[^ ^20^ ^]^ multiplex immunofluorescence (mIHC) staining was performed, revealing a significant correlation between CCT6A and FAP expression (Figure 1D). Quantitative analysis revealed higher CCT6A‐positive cell frequency in CAFs versus NFs (*p* < 0.001, Figure 1E) but not epithelium (*p*  =  0.389). Both CCT6A‐positive cell proportion (*r = 0.485*, *p* < 0.001) and staining intensity (*r = 0.334*, *p* < 0.001, Figure 1F) correlated with CAF abundance, establishing a CAF‐specific expression pattern in GC.

To evaluate the clinical significance of CCT6A‐positive CAFs, we compared chemo‐resistant (RPC, refractory to chemotherapy) and non‐RPC patient groups. The results revealed a significantly higher enrichment of CCT6A‐positive CAFs and serum CCT6A levels in the RPC group compared to the non‐RPC group (*p* < 0.001, Figure 1G) and (*p* < 0.001, Figure 1H). These findings position CCT6A as both a predictive biomarker for chemotherapy response and a potential therapeutic target in treatment‐resistant GC.

Among 498 GC patients, 67 underwent preoperative PET/CT imaging (Figure 1I). Of these, 36 samples were classified as high‐CCT6A immunostaining (based on the mean percentage of CCT6A‐positive cells). These high‐CCT6A tumors demonstrated significantly elevated SUVmax value, a measure of tumor lesion metabolic activity, in both epithelial cells (*p* = 0.016, Figure 1J) and CAFs (*p* = 0.010, Figure 1J), directly linking CCT6A expression to enhanced glycolytic activity in GC.

Furthermore, survival analysis demonstrated that while CCT6A‐positive epithelial cells showed no significant correlation with the clinicopathological features, CCT6A‐positive CAFs correlated with vascular invasion (*p* = 0.008), Lauren classification (*p* = 0.007), T stage (*p* = 0.003), N stage (*p* = 0.049), M stage (*p* = 0.003), and TNM stage (*p* = 0.002) (Table S1, Supporting Information). Kaplan–Meier analysis revealed that patients with a high abundance of CCT6A‐positive CAFs exhibited significantly worse overall survival (OS, *p* < 0.001) and disease‐free survival (DFS, *p* = 0.001, Figure 1K) compared with patients with low abundance of CCT6A‐positive CAFs. Similar trends were observed in patients with recurrent or metastatic disease (OS, *p* = 0.025 and DFS, *p* = 0.015, Figure 1L). Univariate and multivariate Cox regression analyses demonstrated that the abundance of CCT6A‐positive CAFs, along with M stage and TNM stage, were independent prognostic factors for both OS and DFS in GC patients (Tables S2 and S3, Supporting Information). These results establish stromal CCT6A as a biomarker of GC aggressiveness, chemoresistance, and poor outcomes.

### 2.2 CCT6A Promotes β‐Catenin Nuclear Translocation via Phosphorylation Regulation

**Figure 2:**
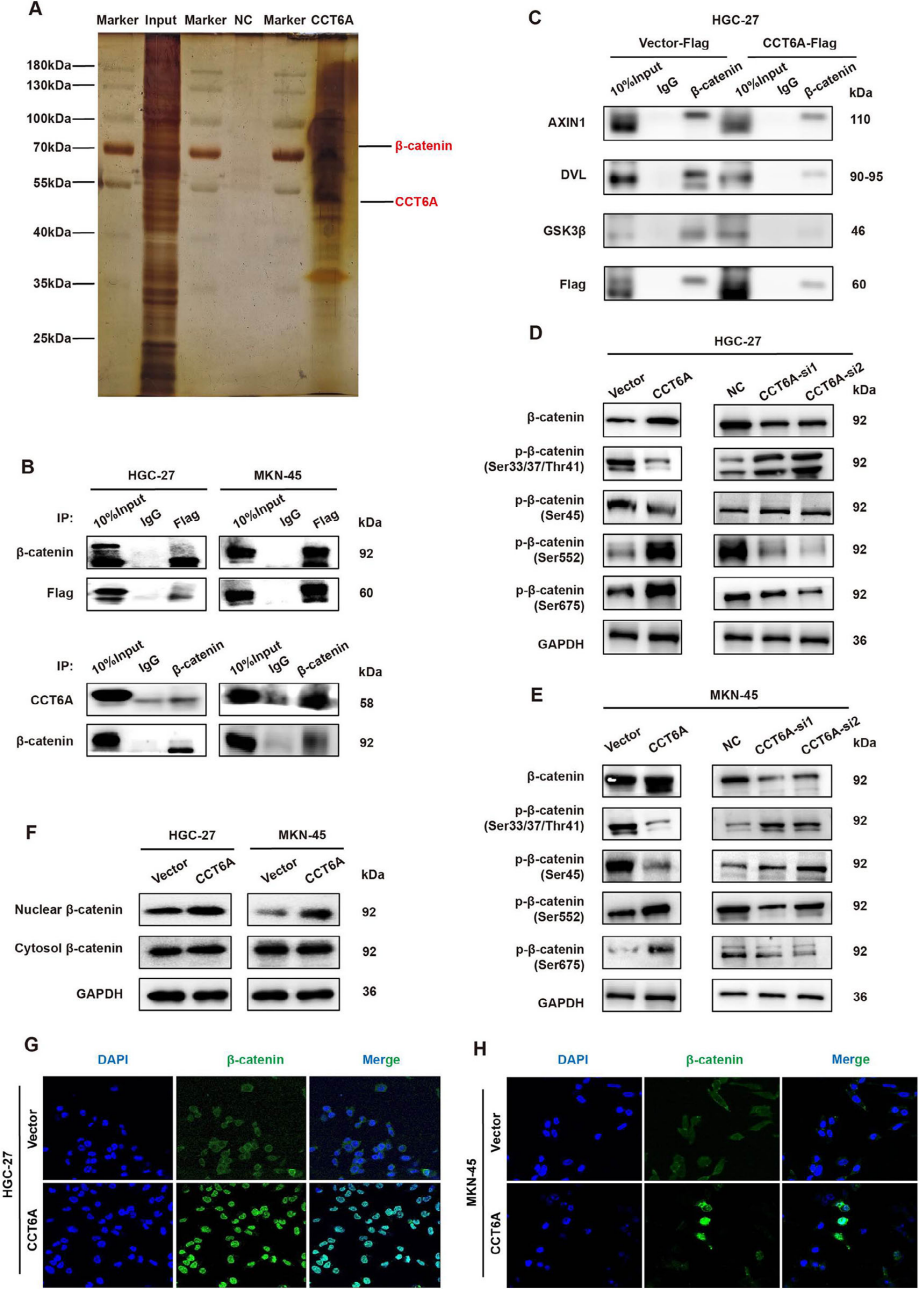
CCT6A mediates β‐catenin phosphorylation and nuclear translocation via direct interaction. A) Immunoprecipitation (IP) of Flag‐tagged CCT6A in HEK293T cells, followed by silver staining to identify interacting proteins. B) Co‐immunoprecipitation (Co‐IP) assays in HGC‐27 and MKN‐45 cells co‐cultured with CAFs and expressing Flag‐tagged CCT6A. C) Co‐IP analysis of β‐catenin interaction with endogenous AXIN1, DVL, and GSK3β in HGC‐27 cells co‐cultured with CAFs upon CCT6A overexpression. D,E) Western blot analysis of active β‐catenin and phosphorylation status (Ser33/37/Thr41, Ser45, Ser552, Ser675) in HGC‐27 D) and MKN‐45 E) cells co‐cultured with CAFs after CCT6A knockdown or overexpression. F–H) Subcellular fractionation F) and immunofluorescence G,H) demonstrating β‐catenin nuclear localization in HGC‐27 and MKN‐45 cells co‐cultured with CAFs following CCT6A overexpression.

To investigate CCT6A‐mediated oncogenic signaling, we performed Flag‐tagged affinity purification coupled with LC‐MS/MS, identifying β‐catenin as a primary interacting partner (**Figure** **2**A). Among all co‐precipitated proteins, β‐catenin was selected for further validation due to its high coprecipitation levels compared to the negative control. Endogenous co‐immunoprecipitation (co‐IP) assays confirmed direct CCT6A‐β‐catenin binding (Figure 2B). Mechanistically, CCT6A competitively disrupts β‐catenin's interaction with GSK3β and AXIN1, thereby stabilizing β‐catenin by blocking its degradation (Figure 2C).

To delineate CCT6A‐mediated regulation of β‐catenin, we performed gain‐ and loss‐of‐function assays in HGC‐27 and MKN‐45 cells, demonstrating that CCT6A overexpression elevated total β‐catenin levels, whereas its depletion reduced them (Figure 2D,E).

β‐catenin, a central Wnt pathway effector, undergoes context‐dependent phosphorylation: 1) CK1 (Ser45) and GSK‐3β (Ser33/37/Thr41) prime it for degradation,^[^ ^21^, ^22^ ^]^ while 2) AKT (Ser552) and PKA (Ser675) enhance its stability and transcriptional activity.^[^ ^23^ ^]^ Phosphorylation profiling revealed that CCT6A promoted β‐catenin phosphorylation at Ser552 and Ser675, thereby stabilizing β‐catenin and enhancing its transcriptional activity. Concurrently, CCT6A inhibited phosphorylation at Ser33, Ser37, Thr41, and Ser45, which would normally target β‐catenin for degradation (Figure 2D,E). Immunofluorescence and immunoblotting confirmed CCT6A‐driven β‐catenin nuclear accumulation in HGC‐27 and MKN‐45 cells (Figure 2F–H). These results suggest that CCT6A activates Wnt signaling by stabilizing β‐catenin, facilitating its nuclear translocation, and enhancing its transcriptional activity.

### 2.3 CCT6A/β‐Catenin Interaction Represses DDIT4 and TXNIP Transcription by Enhancing c‐Myc Activity

**Figure 3:**
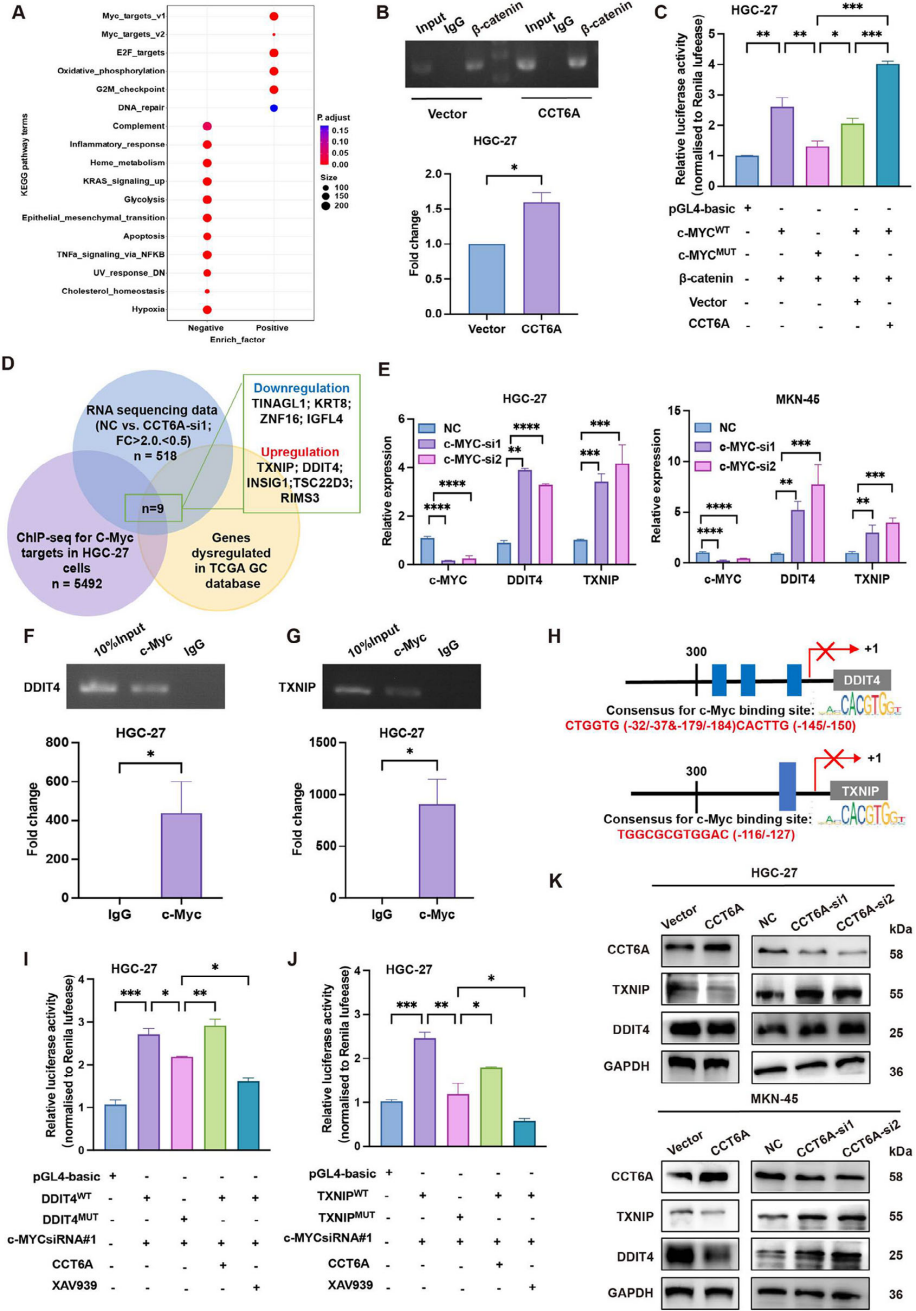
The CCT6A/β‐catenin/c‐Myc axis suppresses DDIT4 and TXNIP transcription. A) Pathway analysis of differentially expressed genes in HGC‐27 cells co‐cultured with CCT6A‐knockdown CAFs versus controls. B,C) Chromatin immunoprecipitation (ChIP) and luciferase reporter assays demonstrating enhanced β‐catenin recruitment to the c‐Myc promoter in CCT6A‐overexpressing HGC‐27 cells. D) Integrated analysis of ChIP‐seq, RNA‐seq, and TCGA data revealing dysregulated c‐Myc target genes. E) RT‐qPCR analysis of mRNA levels of TXNIP and DDIT4 in HGC‐27 and MKN‐45 cells with c‐Myc knockdown. F‐G) ChIP assays confirming c‐Myc binding to TXNIP and DDIT4 promoters, quantified by RT‐qPCR and normalized to IgG. H) Schematic of DDIT4 and TXNIP reporter constructs, with consensus (blue) and putative c‐Myc binding sites indicated. I,J) Luciferase reporter assays evaluating DDIT4 and TXNIP promoter activity upon modulation of c‐Myc siRNA#1, CCT6A overexpression, β‐catenin inhibitor XAV939. K) Western blot analysis showing CCT6A overexpression reduces, while CCT6A knockdown elevates, DDIT4 and TXNIP protein levels in HGC‐27 and MKN‐45 cells. (Data represent mean±SEM. *p*‐values calculated by Mann–Whitney U‐test or one‐way ANOVA. ns, not significant; ^*^p < 0.05; ^**^p < 0.01; ^***^p < 0.001; ^****^p < 0.0001).

Pathway analysis of CCT6A‐knockdown GC cells revealed significant enrichment of Myc target genes, implicating c‐Myc may as a key downstream effector of CCT6A/β‐catenin signaling (**Figure** **3**A). Based on established β‐catenin‐mediated c‐Myc activation, we hypothesized that CCT6A enhances β‐catenin recruitment to the c‐Myc promoter, thereby amplifying its transcriptional activity.^[^ ^24^ ^]^ Chromatin immunoprecipitation (ChIP) and luciferase reporter assays in HGC‐27 cells confirmed that CCT6A overexpression markedly enhanced β‐catenin binding at the c‐Myc promoter (Figure 3B,C).

To delineate c‐Myc targets in CCT6A‐mediated signaling pathway, we performed ChIP‐sequencing (ChIP‐seq) data with RNA‐seq from CCT6A‐knockdown GC cells and TCGA GC datasets (dysregulated genes vs. normal tissues), identifying nine conserved c‐Myc target genes (Figure 3D). Among these, DNA damage‐inducible transcript 4 (DDIT4) and Thioredoxin Interacting Protein (TXNIP) were further validated as direct transcriptional targets by RT‐qPCR (Figure 3E; Figure S1B, Supporting Information). ChIP‐qPCR analysis confirmed the presence of c‐Myc binding sites within the promoter regions of DDIT4 and TXNIP (Figure 3F,G).

Predicted c‐Myc binding sites from JASPAR (http://jaspar.genereg.net/) were further tested using luciferase reporter assays. Reporter plasmids containing wild‐type (WT) and mutated (MUT) c‐Myc binding sites showed that DDIT4 and TXNIP promoter activity increased in c‐Myc‐knockdown cells, an effect that was abolished upon mutation of the predicted c‐Myc binding sites. Furthermore, CCT6A overexpression restored c‐Myc‐mediated repression, which was further enhanced by treatment with the β‐catenin inhibitor XAV939 (Figure 3H–J). Western blot analysis revealed that overexpression of CCT6A significantly suppressed DDIT4 and TXNIP protein levels (Figure 3K). Collectively, these findings establish a CCT6A/β‐catenin/c‐Myc axis that transcriptionally repression DDIT4 and TXNIP to drive GC progression.

### 2.4 CCT6A is a ceRNA Target of the CCT6P1/miR‐922 Interaction

Sequence analysis identified CCT6P1, a highly homologous hsp60 pseudogene containing tandem duplications of the CCT6A RNA sequence (Figure S1C, Supporting Information). Both CCT6A (Figure 1A) and CCT6P1 (Figure S1D–F, Supporting Information) were significantly upregulated in GC tissues compared to normal gastric mucosa in the TCGA‐GC and FUSCC RT‐qPCR cohorts. Correlation analysis revealed a positive correlation between CCT6P1 and CCT6A expression in the TCGA GC cohort (*r = 0.244, p* < 0.001, Figure S1G, Supporting Information) and the Cancer Cell Line Encyclopedia (CCLE) GC cells cohort (*r = 0.401, p*  =  0.014, Figure S1H, Supporting Information). Although no significant correlation was observed at the mRNA level in the FUSCC RT‐PCR cohort (*r = ‐0.133, p*  =  0.416, Figure S1I, Supporting Information), CCT6P1 was positively associated with CCT6A‐positive epithelial cells in the FUSCC mIHC cohort (*r = 0.334, p*  =  0.040, Figure S1J, Supporting Information).

**Figure 4:**
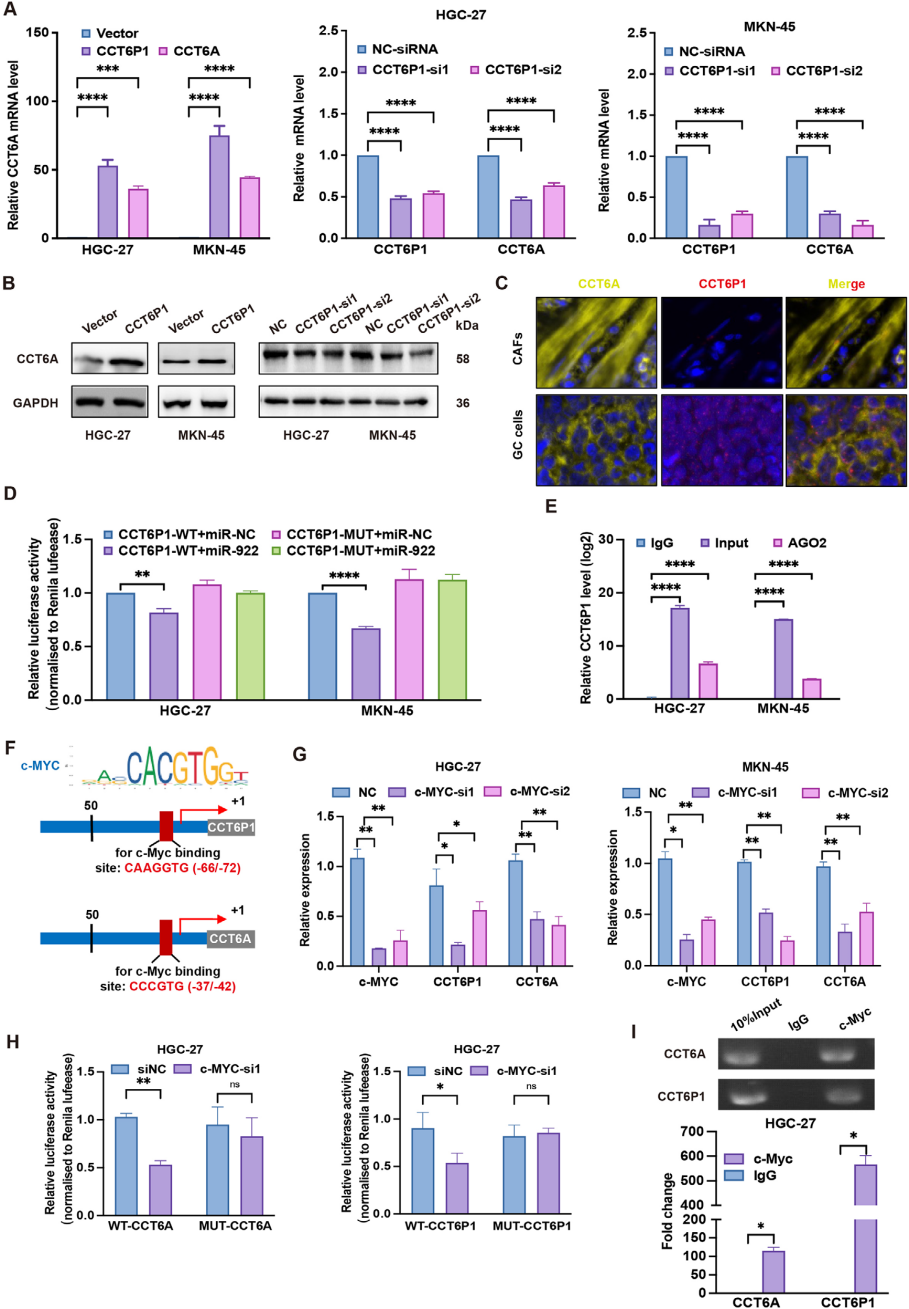
CCT6A function as a ceRNA regulated by CCT6P1/miR‐922, with transcriptional control by c‐Myc. A,B) RT‐qPCR and western blot analysis of CCT6A expression in HGC‐27 and MKN‐45 cells following CCT6P1 overexpression or knockdown. C) mIHC showing spatial co‐localization of CCT6P1 RNA and CCT6A protein. D) Luciferase reporter assays comparing wild‐type (WT) versus mutated (Mut) CCT6P1 constructs in cells transfected with miR‐922 mimic in HGC‐27 and MKN‐45 cells. E) Anti‐Ago2 RIP was used to pull down endogenous RNAs associated with Ago2, IgG served as the control. The levels of CCT6P1 were measured by RT‐qPCR, and the data are presented as fold enrichment in Ago2 relative to input. F) Schematic of CCT6P1 and CCT6A reporter constructs, with c‐Myc binding sites (red boxes) and putative sequences indicated. G) RT‐qPCR analysis of CCT6P1 and CCT6A mRNA levels upon c‐Myc knockdown in HGC‐27 and MKN‐45 cells. H) Luciferase assays of WT versus mutant CCT6A/CCT6P1 promoters in c‐Myc‐silenced cells. I) ChIP‐qPCR assays demonstrated that c‐Myc directly occupied the predicted binding sites of both CCT6A/CCT6P1 promoters. (Data represent mean ± SEM. *p*‐values calculated by one‐way ANOVA and Mann–Whitney U‐test. ns, not significant; ^*^p < 0.05; ^**^p < 0.01; ^***^p < 0.001; ^****^p < 0.0001).

Gain‐ and loss‐of‐function experiments showed that CCT6P1 overexpression increased, while its knockdown reduced, CCT6A mRNA and protein levels in GC cells (**Figure** **4**A,B). Subcellular fractionation and mIHC analysis revealed that CCT6P1 RNA predominantly localized in the cytoplasm and co‐localized with CCT6A protein, suggesting post‐transcriptional regulation (Figure 4C; Figure S1K, Supporting Information). Based on its cytoplasmic location and known ceRNAs mechanisms, we propose CCT6P1 competitively regulates CCT6A through miRNA sequestration.

RNA sequencing in GC cells following CCT6P1 knockdown identified 156 upregulated miRNAs compared to the negative control. Bioinformatics analysis using miRNA target prediction tools (miRabel) and intersection with RNA sequencing results revealed 13 miRNAs that shared consensus binding sites in the 3′UTR of both CCT6P1 and CCT6A. Among them, five miRNAs (miR‐616‐5p, miR‐590‐3p, miR‐25‐3p, miR‐922 and miR‐199a‐5p) were significantly downregulated in CCT6P1‐overexpressing GC cells (Figure S2A,B, Supporting Information). Notably, only miR‐922 consistently suppressed CCT6A expression at both mRNA and protein levels in GC cells (Figure S2C,D, Supporting Information). In addition, miR‐922 was inversely correlated with CCT6P1 levels, increasing upon CCT6P1 knockdown and decreasing with CCT6P1 overexpression (Figure S2E, Supporting Information). Although transfection of miR‐922 mimics in GC cells has no effect on CCT6P1 expression (Figure S2F,G, Supporting Information), it partially reversed the CCT6A upregulation induced by CCT6P1‐overexpression (Figure S2H, Supporting Information).

We further validated the direct interaction between CCT6P1 and miR‐922 using a dual luciferase reporter assay. The results demonstrated that miR‐922 mimics significantly reduced the luciferase activities of the reporter plasmid containing the wild‐ type (WT) binding sites of CCT6P1, but had no effect on the mutated sequence (mutant type, MUT; Figure 4D; Figure S2I, Supporting Information), indicating that miR‐922 directly binds to CCT6P1. RNA immunoprecipitation (RIP) assays using an anti‐Ago2 antibody further validated that CCT6P1 and miR‐922 were enriched in the Ago2 complex, indicating functional miRNA interaction (Figure 4E).

In addition, co‐transfection of miR‐922 mimics effectively rescued CCT6A overexpression induced by CCT6P1, supporting a ceRNA mechanism (Figure S2H,J, Supporting Information), implying that CCT6A may be a target of the CCT6P1/miR‐922 interaction in GC. Similarly, miR‐922 directly targeted the 3′UTR of CCT6A, as confirmed by luciferase assays and Ago2 RIP, which enriched CCT6A and miR‐922 in HGC‐27 and MKN‐45 cells (Figure S2K,L, Supporting Information). Furthermore, co‐transfection of miR‐922 mimics attenuated CCT6A protein level, even in the presence of CCT6A overexpression (Figure S2M, Supporting Information). Taken together, these results indicate that CCT6P1 may function as a miR‐922 sponge, stabilizing CCT6A expression via a ceRNA mechanism, thereby contributing to GC progression.

### 2.5 CCT6A and CCT6P1 are Direct Transcriptional Targets of c‐Myc

To investigate the regulatory mechanisms underlying CCT6P1 and CCT6A overexpression in GC, we performed promoter analysis (≈ 2 kb upstream) using UCSC and JASPAR, identifying conserved c‐Myc binding motifs (CCACCTG/C) in both genes' promoters (Figure 4F). Functional validation revealed that c‐Myc overexpression significantly upregulated CCT6P1 and CCT6A, while c‐Myc knockdown reduced their expression (Figure 4G). To confirm direct transcriptional regulation, wild‐type and mutant promoter sequences were cloned into luciferase reporter constructs. As expected, ectopic c‐Myc expression enhanced luciferase activity of the wild‐type constructs but had no effect on the mutant versions (Figure 4H). ChIP‐qPCR assays further demonstrated that c‐Myc directly occupied the predicted binding sites of both promoters (Figure 4I). These findings establish a c‐Myc‐driven transcriptional circuit that amplifies CCT6P1/CCT6A expression to sustain oncogenic signaling in GC.

### 2.6 CCT6A Enhances CAF Proliferation, Glycolysis, and Transport to GC Cells via Extracellular Vesicles

**Figure 5:**
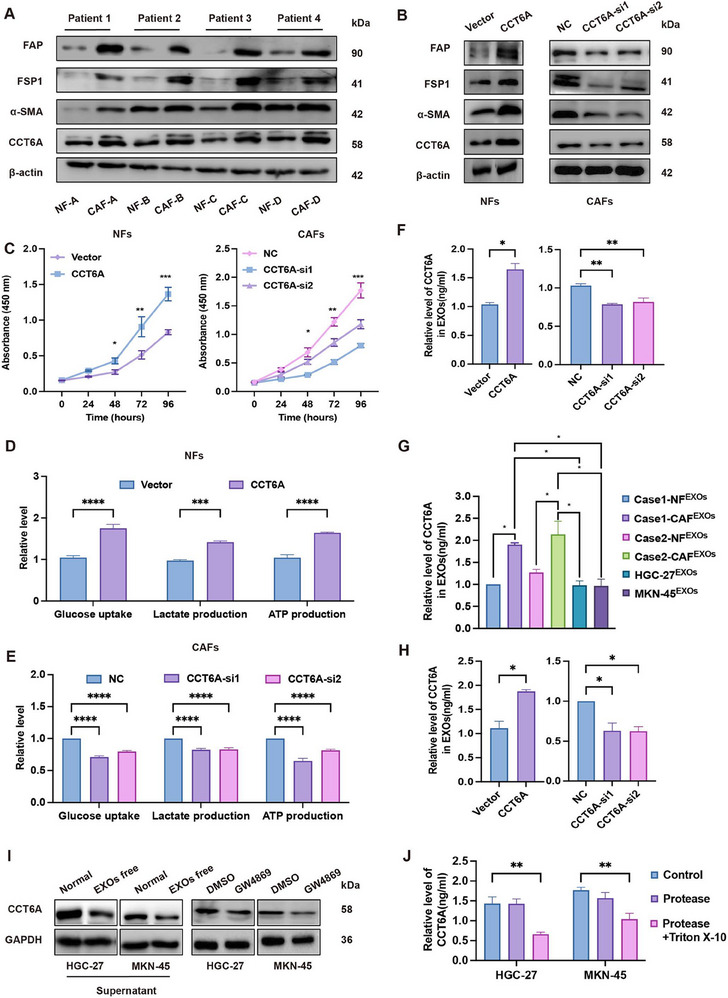
CCT6A regulates CAF proliferation, glycolysis, and transport to GC cells via extracellular vesicles. A) Western blot analysis of CCT6A, FSP1, α‐SMA and FAP expression in NFs and CAFs from GC. B) Western blot showing CCT6A, FSP1, α‐SMA and FAP expression in NFs with CCT6A overexpression or CAFs with CCT6A knockdown. C) Cell proliferation assays in NFs transfected with CCT6A overexpression plasmid (vs empty vector) and CAFs transfected with CCT6A‐targeting siRNAs (vs control siRNA). D,E) Glycolytic activity measured by glucose uptake, ATP production, and lactate generation in NFs with CCT6A overexpression D) and CAFs with CCT6A knockdown E). F) ELISA results showing the level of CCT6A protein in the supernatant of NFs and CAFs after CCT6A overexpression in NFs or knocking down in CAFs. G) ELISA results showing the level of CCT6A protein in the supernatant exosomes of NFs, CAFs, and human GC cells HGC‐27 and MKN‐45. H) ELISA results showing the level of CCT6A protein in the supernatant exosomes of CAFs after CCT6A overexpressing in NFs or knockdown in CAFs. I,J) Western blot I) and ELISA J) showing reduced CCT6A levels in co‐cultured HGC‐27 and MKN‐45 cells after exosome depletion from CAF‐conditioned medium. (Data represent mean ± SEM. p‐values calculated by two‐way ANOVA, Mann–Whitney U‐test and one‐way ANOVA. ns, not significant; ^*^p < 0.05; ^**^p < 0.01; ^***^p < 0.001; ^****^p < 0.0001).

Given the strong CCT6A immunostaining observed in GC‐CAFs, we characterized its functional significance in these cells. Primary CAFs and NFs were isolated from fresh GC and adjacent non‐tumor tissues. Immunofluorescence analysis revealed that CAFs exhibited higher levels of fibroblast activation protein (FAP), Vimentin and alpha‐smooth muscle actin (α‐SMA) compared to NFs (Figure S3A, Supporting Information). Immunoblotting further confirmed significantly higher CCT6A expression in CAFs compared to NFs (**Figure** **5**A). Overexpression of CCT6A in NFs promoted upregulation of FAP, FSP1, andα‐SMA levels, while CCT6A knockdown in CAFs resulted in the opposite effect (Figure 5B). Additionally, modulation of CCT6A expression in both NFs and CAFs correspondingly alters their proliferation(Figure 5C). Given the positive correlation between CCT6A and glucose metabolism, we examined its role in CAF glycolysis. Overexpression of CCT6A in NFs enhanced glucose uptake, lactate production, and ATP generation, while knockdown of CCT6A in CAFs had the opposite effect (Figure 5D,E).

ELISA assays further confirmed that exosomal CCT6A protein levels in the supernatant of CAFs were modulated by CCT6A expression (Figure 5F). Although previously detected in glioblastoma‐derived vesicles ^[^ ^16^ ^]^CCT6A's presence in GC‐CAFs exosomes was uncharacterized. Using transmission electron microscopy (Figure S3B, Supporting Information), nanoparticle tracking analysis (Figure S3C, Supporting Information), and Western blot (Figure S3D, Supporting Information), we successfully extracted extracellular vesicles secreted by NFs, CAFs, and human GC cells (HGC‐27 and MKN‐45). Immunofluorescence analysis demonstrated that PHK67‐labeled CAF exosomes (CAF^EXOs^) were efficiently internalized by GC cells (Figure S3E, Supporting Information). Notably, ELISA revealed significantly higher CCT6A levels in CAF^EXOs^ than those in paired NF^EXOs^, or exosomes derived from human GC cells (HGC‐27 and MKN‐45) (Figure 5G). Furthermore, modulation of CCT6A expression in CAFs similarly altered its abundance in secreted exosomes (Figure 5H). These results indicate that CCT6A is highly expressed in extracellular vesicles derived from CAFs.

To investigate whether CAFs transfer CCT6A to GC cells via exosomes, we depleted exosomes from CAF‐conditioned medium using ultracentrifugation and the exosome inhibitor GW4869. Both methods significantly reduced CCT6A levels in co‐cultured GC cells (Figure 5I). Additionally, ELISA experiments showed no significant change in CCT6A levels in the supernatant of CAFs before and after protease treatment to remove free protein molecules (Figure 5J). However, when Triton X‐100 was added to disrupt the exosome membranes, followed by protease treatment, a significant decrease in detectable CCT6A protein levels was observed, indicating that CCT6A primarily resides within exosomes rather than in a free extracellular form (Figure 5J). Collectively, these results confirmed that CCT6A is primarily packaged in exosomes and is transported from CAFs to GC cells via exosomes.

### 2.7 CAF‐Derived Exosomal CCT6A Enhances Stemness, Chemoresistance, and Glycolysis in GC by Activating β‐catenin/DDIT4‐TXNIP Axis

**Figure 6:**
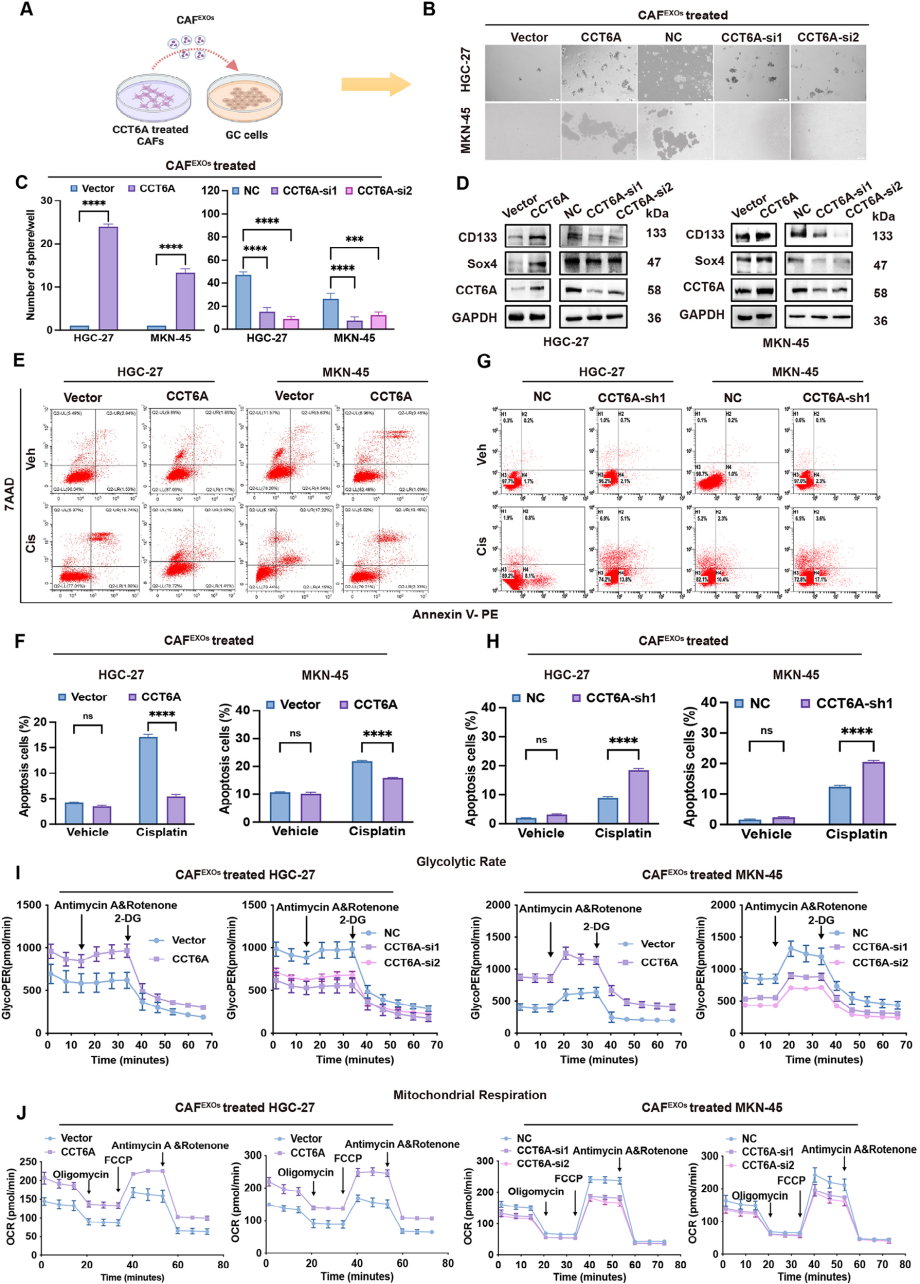
CAF‐derived exosomal CCT6A promotes stemness, chemoresistance, and glycolysis in Gastric cancer. A) Schematic of the co‐culture experimental design. B,C) Sphere formation assays demonstrating stemness properties of HGC‐27 and MKN‐45 cells treated with exosomes (EXOs) from CCT6A knockdown or overexpression CAF‐derived exosomes. D) Western blot analysis of stemness markers (CD133, SOX4) and CCT6A in GC cells treated with CCT6A knockdown or overexpression CAF EXOs. E‐H) Flow cytometry analysis showing the proportion of apoptotic cells in HGC‐27 and MKN‐45 cells treated for 24h with CCT6A knockdown or overexpression CAF EXOs or CAFs followed by cisplatin treatment. I,J) Seahorse extracellular flux analysis measuring the glycolytic proton efflux rate (GlycoPER) and oxygen consumption rate (OCR) in HGC‐27 and MKN‐45 cells treated with CCT6A knockdown or overexpression CAF EXOs or CAFs following by cisplatin treatment. (Data represent mean ± SEM. *p*‐values calculated by Mann–Whitney U‐test and one‐way ANOVA. ns, not significant; ^*^p < 0.05; ^**^p < 0.01; ^***^p < 0.001; ^****^p < 0.0001).

Given the established link between Wnt/β‐catenin signaling and cancer cell stemness, we investigated whether CAF‐derived exosomal CCT6A regulates GC cell stemness, chemoresistance, and glycolysis in GC cells. HGC‐27 and MKN‐45 cells were treated with CAF^EXOs^ that derived from CCT6A overexpressing or knockdown CAFs, along with their respective controls (Vector or NC), prior to in vitro functional assay (**Figure** **6**A). Sphere formation assays revealed that CCT6A‐overexpressing CAF^EXOs^ enhanced, while CCT6A‐knockdown CAF^EXOs^ suppressed, the self‐renewal capacity of GC cells (Figure 6B–D). Additionally, cancer cells co‐incubated with CCT6A‐overexpressing CAF^EXOs^ exhibited reduced apoptosis upon cisplatin treatment, whereas those treated with CCT6A‐knockdown CAF^EXOs^ showed significantly increased apoptosis (Figure 6E–H).

Metabolic profiling further demonstrated that CCT6A‐overexpressing CAF^EXOs^ promoted glycolysis and oxidative phosphorylation in GC cells. This was evidenced by increased expression of rate‐limiting glycolytic enzymes (Figure S4A, Supporting Information), enhanced glucose uptake, lactate and ATP production (Figure S4B, Supporting Information), as well as elevated glycolytic proton efflux rate (GlycoPER; reflects glycolysis) and oxygen consumption rate (OCR; reflects mitochondrial respiration) (Figure 6I,J; Figure S4C,D, Supporting Information).

**Figure 7:**
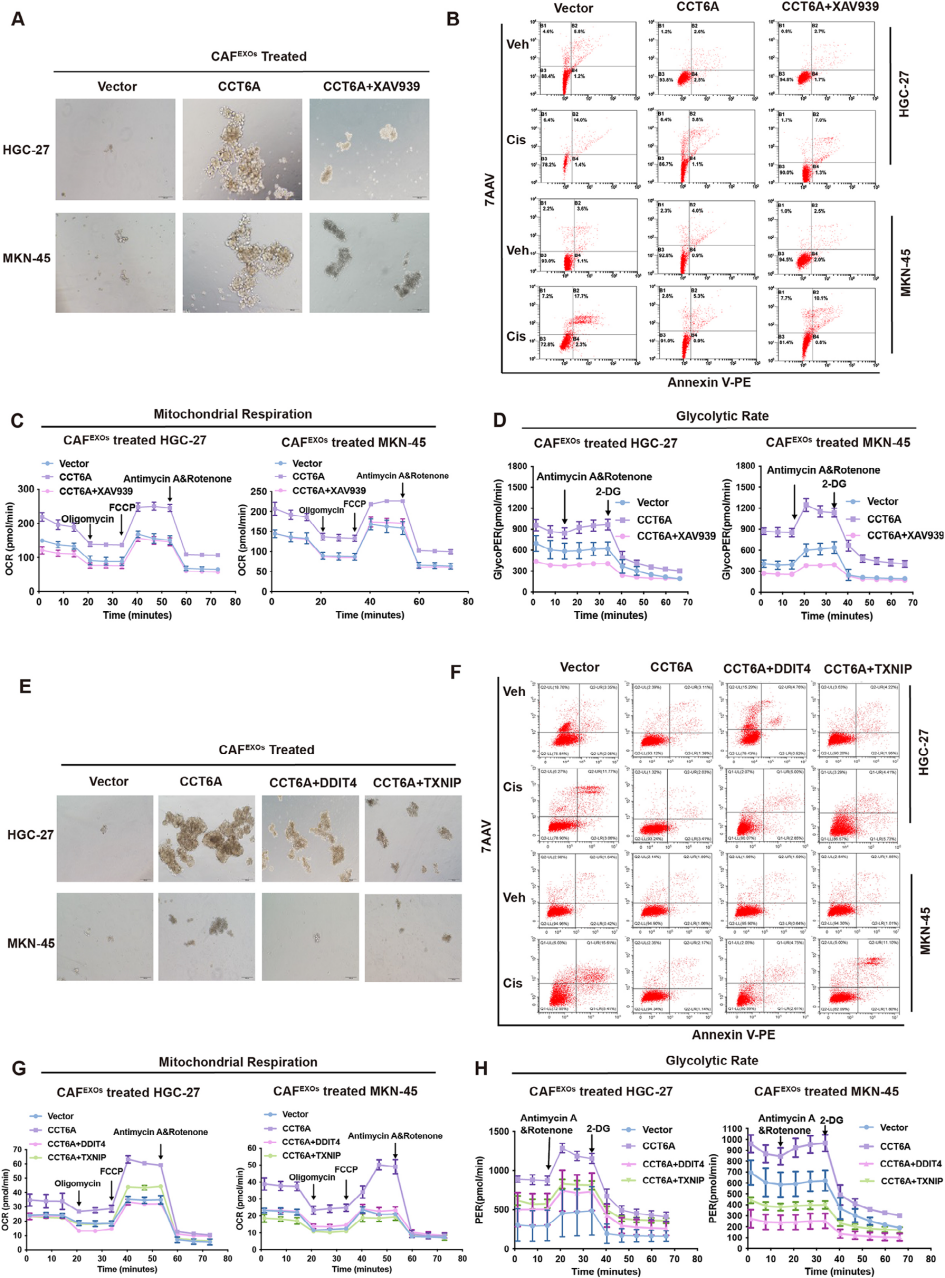
β‐catenin inhibition or DDIT4/TXNIP overexpression reverses CAF‐derived exosomal CCT6A on stemness, chemoresistance, and glycolysis in GC. A–D) Spheroid formation assay, flow cytometry, and seahorse extracellular flux analysis demonstrating spheroid formation capacity, apoptotic cell proportion, glycolytic proton efflux rate (GlycoPER), and oxygen consumption rate (OCR) in HGC‐27 and MKN‐45 cells treated with CCT6A vector CAF EXOs, CCT6A‐overexpressing CAF EXOs, or CCT6A‐overexpressing CAF EXOs combined with β‐catenin inhibitor XAV939. E‐H) Spheroid formation assay, Flow cytometry, and Seahorse extracellular flux analysis showing spheroid formation ability, proportion of apoptotic cells, GlycoPER, and OCR in HGC‐27 and MKN‐45 cells treated with CCT6A vector CAF EXOs, CCT6A‐overexpressing CAF EXOs, and co‐transfected with DDIT4 or TXNIP.

Furthermore, treatment with the Wnt/β‐catenin signaling inhibitor XAV939 significantly inhibited GC cell stemness, chemoresistance, and glucose metabolism in CCT6A^+^ CAF‐treated cells (**Figure** **7**A–D; Figure S4E–G, Supporting Information). To explore whether the DDIT4‐TXNIP axis mediates the effects of CAF‐derived CCT6A on cancer cell malignancy in GC, we overexpressed DDIT4 and TXNIP in HGC‐27 and MKN‐45 cells via plasmid transfection (Figure S4H, Supporting Information). Notably, overexpression of DDIT4 and TXNIP in HGC‐27 and MKN‐45 cells partially reversed the pro‐tumorigenic effects of CCT6A‐overexpressing CAF^EXOs^, reducing sphere formation efficiency, cisplatin resistance, and metabolic activity (Figure 7E–H; Figure S4I–K, Supporting Information). Taken together, these results suggest that CCT6A promotes GC cell malignancy, at least in part, through activation of the β‐catenin/DDIT4‐TXNIP axis.

**Figure 8:**
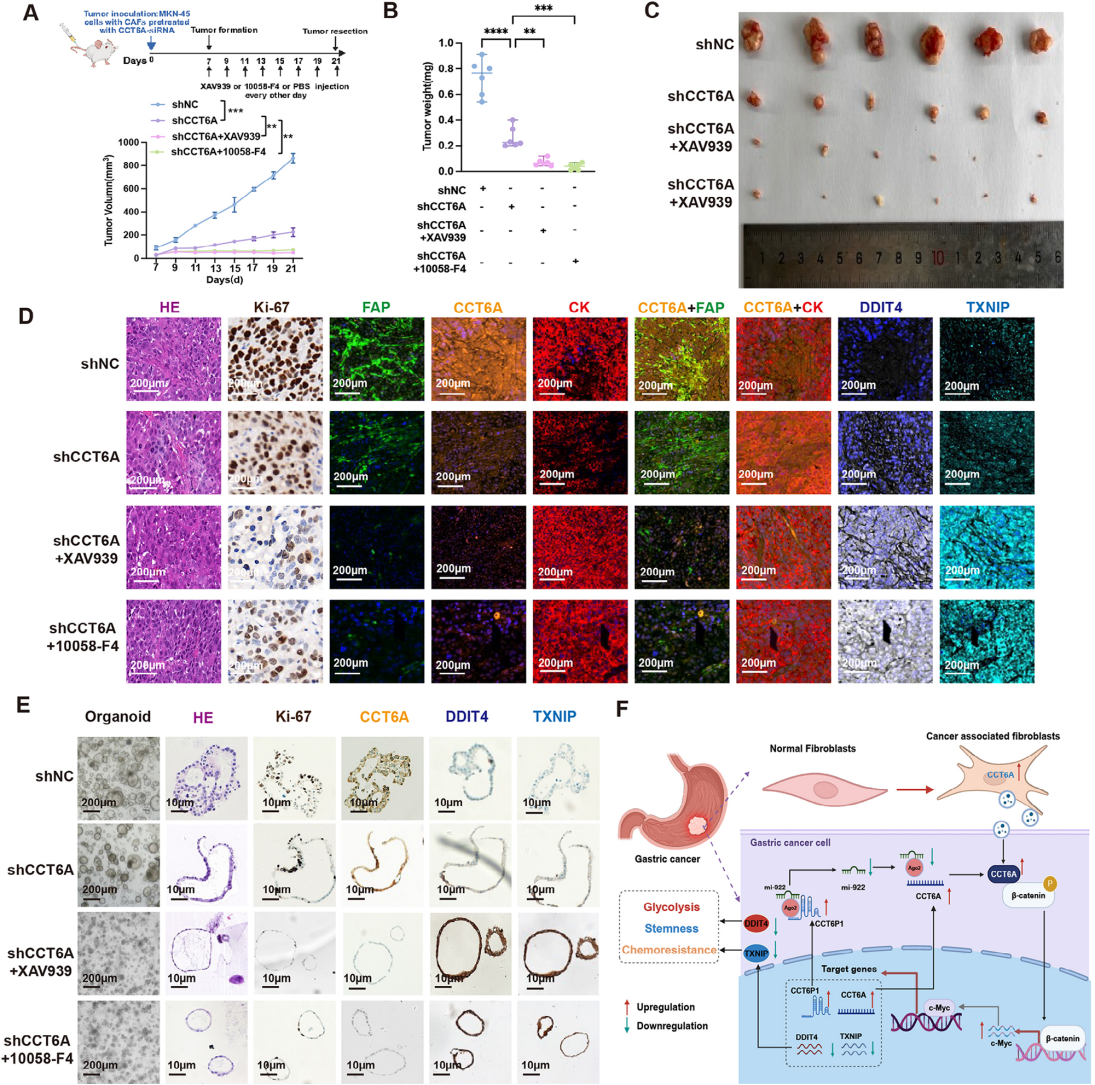
Inhibition of β‐catenin/c‐Myc axis synergizes with CCT6A knockout to suppress tumor growth in xenograft and organoid models. A) Schematic diagram of the vivo animal experiment (top) and tumor growth curve (bottom). MKN‐45 cells with stable knockdown of CCT6A were subcutaneously implanted into nude mice. β‐catenin inhibitor (XAV939, 20 mg kg^−1^) and c‐Myc inhibitor (10058‐F4,15 mg kg^−1^) were administered intraperitoneally (every 2 days, for a total of eight doses) from day 7 to day 21. B,C) Photograph of dissected xenografts before and after treatment. D) Representative images showing H&E and mIHC staining for Ki‐67, FAP, CCT6A, CK, CCT6A+FAP, CCT6A+CK. DDIT4 and TXNIP in tumor tissue from the indicated groups. Scale bar = 200 µm. E) Bright‐field images (Scale bar = 200 µm), H&E and mIHC (Scale bar = 10 µm) staining of GC organoids for Ki‐67, FAP, CCT6A, DDIT4, and TXNIP. F) Mechanistic diagram summarizing the findings of this study. Figures created with BioRender (https://biorender.com/). (Data represent mean ± SEM. *p*‐values calculated by two‐way ANOVA and one‐way ANOVA. ns, not significant; ^*^p < 0.05; ^**^p < 0.01; ^***^p < 0.001; ^****^p < 0.0001).

To further assess the therapeutic potential of targeting the Wnt/β‐catenin/c‐Myc signaling pathway in CCT6A‐knockdown CAFs, mice were xenografted with control or CCT6A‐knockdown MKN‐45 cells. Seven days after injection of the CCT6A‐knockdown MKN‐45 cells, two groups of mice were peritumorally injected every other day with XAV‐939 (20 mg kg^−1^) or the c‐Myc inhibitor 10058‐F4 (15 mg kg^−1^) for 2 weeks (**Figure** **8**A). As expected, tumor growth was significantly reduced in the CCT6A‐knockdown group, with further suppression observed upon treatment with XAV939 or 10058‐F4 (Figure 8A–C). Additionally, patient‐derived organoid (PDO) models established from GC tissues confirmed that CCT6A knockdown suppressed PDO formation (Figure 8E). IHC analysis of xenografted tumors and PDO tissues further supported the role of CAF‐derived exosomal CCT6A in tumor progression, as evidenced by decreased Ki‐67 expression and increased expression of DDIT4 and TXNIP, modulated via the β‐catenin/c‐Myc axis (Figure 8D,E). These findings highlight the critical role of targeting key components of the CCT6A/β‐catenin/c‐Myc axis in modulating GC biology and offer potential therapeutic insights for GC treatment.

## 3 Discussion

GC remains persists as a leading cause of cancer‐related mortality worldwide, with late‐stage diagnosis significantly limiting curative treatment options. While perioperative chemotherapy remains the standard approach for resectable locally advanced GC, postoperative recurrence and distant metastasis frequently occur despite surgical intervention.^[^ ^2^ ^]^ The TME, particularly CAFs, plays a crucial role in GC progression. In this study, we demonstrate that CAF‐derived exosomal CCT6A promotes stemness, chemoresistance, and glycolysis in GC cells through activation of β‐catenin/c‐Myc/DDIT4‐TXNIP axis. Furthermore, we identify CCT6P1, a pseudogene of CCT6A, acts as a ceRNA that modulates CCT6A expression by sponging miR‐922. In addition, c‐Myc was found to transcriptionally upregulate both nuclear CCT6P1 and CCT6A in GC cells. Collectively, our findings highlight CCT6A as a promising therapeutic target for CAF‐directed interventions in GC (Figure 8F).

CCT6A is ubiquitously expressed across various tissues but demonstrates cancer‐specific overexpression, positioning it as a potential biomarker and therapeutic target.^[^ ^11^ ^]^ While previous studies have reported significant CCT6A upregulation in epithelial tissue and its association with poor prognosis in GC,^[^ ^19^ ^]^ our study reveals novel insights through mIHC. In the present study, although bulk tissue analysis confirmed elevated CCT6A expression in GC specimens, mIHC quantification showed no significant difference in CCT6A‐positive epithelial cell proportions. This unexpected finding suggests that non‐epithelial cell populations, particularly CAFs, may be the primary source of increased CCT6A expression in GC. Our research systematically explored the clinical relevance of CCT6A expression. We found that high expression of CCT6A in GC epithelium and CAFs is significantly positively correlated with elevated glucose metabolism levels and insensitivity to postoperative chemotherapy in GC patients. Importantly, our findings highlight that CCT6A expression in CAFs, rather than tumor cells, serves as an independent prognostic factor for poor survival, emphasizing its crucial role in driving GC‐promoting processes. Clinically, we demonstrated that elevated levels of CCT6A in peripheral blood can serve as a non‐invasive marker for predicting postoperative chemosensitivity of GC patients. These translational findings highlight CCT6A's dual role as both a mechanistic driver of GC progression and a clinically actionable target.

In current clinical practice, the therapeutic efficacy of postoperative chemotherapy for advanced GC remains suboptimal. This limitation stems primarily from acquired drug resistance, dose‐limiting toxicities, and other factors that impede prolonged chemotherapeutic administration.^[^ ^3^ ^]^ Our study introduces CCT6A as a promising liquid biopsy biomarker that could enable risk‐stratified management of GC patients receiving adjuvant chemotherapy. The non‐invasive measurement of circulating CCT6A levels may help clinicians identify patients who are more likely to derive clinical benefit from chemotherapy. Notably, our discovery of the strong association between elevated CCT6A expression and cisplatin resistance provides a compelling rationale for investigating combination therapies that pair conventional chemotherapy with CCT6A‐targeted agents, particularly in patients exhibiting high serum CCT6A levels. While we observed statistically significant differences between the RPC (responders) and non‐RPC (non‐responders) subgroups, the modest sample size in each cohort necessitates cautious interpretation of these results. Subsequent validation studies employing larger, independent patient populations will be essential to confirm these preliminary findings. These results highlight the translational potential of CCT6A‐directed therapies and provide a foundation for implementing precision medicine approaches in advanced GC treatment paradigms.

In the TME, NFs often undergo a series of phenotypic changes and differential expression of functional genes in response to certain carcinogenic factors, thereby activating into CAFs.^[^ ^4^ ^]^ Activated CAFs typically exhibit enhanced proliferative capacity, synthetic activity, and metabolic function,^[^ ^25^ ^]^ further strengthening their pro‐tumorigenic phenotype. Notably, our study shows that overexpression of CCT6A in NFs can promote the expression of CAF markers, suggesting that CCT6A can induce NFs to activate and transform into CAFs in the GC TME. Exosomes, secreted by various cell types, are critical mediators of intercellular communication and influence tumor malignancy behaviors.^[^ ^26^ ^]^ It has been reported that CCT6A is highly expressed in glioblastoma‐derived extracellular vesicles.^[^ ^16^ ^]^ In the present study, we found that the level of CCT6A in tumor cells is higher than that in CAF. However, it remains unclear whether CCT6A in CAFs is transported to GC cells via CAF‐secreted exosomes and how it modulates GC cells. In the present study, we found that the level of CCT6A in tumor cells is higher than in CAFs. However, it remains unclear whether CCT6A in CAFs is transported to GC cells via CAF‐secreted exosomes and how it modulates GC cells. Here, we show that CAF‐derived CCT6A is a key determinant contributing to stemness, therapy resistance, and glycolysis in human GC cells through exosome‐mediated intercellular communication. Generally, the transfer of molecules from donor cells with lower concentrations to recipient cells with higher concentrations requires overcoming concentration gradient barriers. Vesicles, including exosomes, are excellent carriers that facilitate the movement of molecules, overcoming potential energy barriers. The uptake of exosomes by recipient cells is an active, energy‐consuming process, with the amount of uptake dependent on the recipient cells themselves.^[^ ^27^ ^]^ Consistently, we observed that high expression of CCT6A in CAFs leads to a significant increase in aerobic glycolysis and oxidative phosphorylation levels. Although increased CCT6A expression in CAFs was associated with enhanced metabolic activity, its direct role in promoting exosome biogenesis or secretion remains to be elucidated. Further studies are needed to explore whether metabolic reprogramming of CAFs contributes to exosome output or the efficiency of intercellular communication.

We further demonstrated that CCT6A level is significantly enriched in CAF‐derived exosomes compared to primary GC cells. Using PKH67‐labeled exosome tracking assays, we confirmed the efficient transfer of CAF‐derived exosomal CCT6A into recipient GC cells, establishing its role as a key mediator of GC progression. Functional characterization revealed that exosomal CCT6A potently enhances GC cell stemness, chemoresistance, and glycolysis. Recent studies have highlighted the important role of CCT6A in driving cancer progression and exerting pro‐tumor effects.^[^ ^15^ ^]^ Functioning as a critical modulator of cell growth, CCT6A reduces telomerase activity by inhibiting the TCAB1/TERT interaction, downregulates cyclin D, suppresses Akt pathway activation, and activates TGF‐β/Smad/c‐Myc pathway. ^[^ ^28^, ^29^, ^30^, ^31^ ^]^ Collectively, these actions provide a molecular foundation for tumor survival from various perspectives. Additionally, CCT6A is extensively involved in glucose metabolic remodeling‐related signaling pathways within cancer cells, such as TCAB1/TERT/HK2 axis^[^ ^28^ ^]^ and STAT1/HK2 axis,^[^ ^17^ ^]^ and it enhances cancer stemness by activating Notch and Wnt pathways.^[^ ^32^ ^]^ Our mechanistic studies uncovered that CCT6A physically interacts with β‐catenin in GC cells, triggering Wnt/β‐catenin downstream signaling and consequent transcriptional silencing of two critical metabolic gatekeepers‐TXNIP and DDIT4. TXNIP and DDIT4 are indeed well‐recognized regulators of cellular metabolism, particularly in the context of mTORC1 signaling and glucose homeostasis. TXNIP negatively regulates glucose uptake and dictates metabolic phenotypes‐high TXNIP promotes oxidative phosphorylation, while low TXNIP drives aerobic glycolysis.^[^ ^33^, ^34^ ^]^ TXNIP depletion enhances stemness in hepatocellular carcinoma.^[^ ^35^ ^]^ Similarly, DDIT4 suppression in glioma increases stemness and temozolomide resistance via GLUT3 upregulation.^[^ ^36^ ^]^ Consequently, both TXNIP and DDIT4 act to diminish tumor cell stemness by suppressing glycolysis. Our findings demonstrate that CCT6A‐mediated TXNIP/DDIT4 suppression in CAFs promotes both glycolysis and oxidative phosphorylation, consistent with their established roles in metabolic regulation. Importantly, unlike canonical metabolic regulators in GC (e.g., PI3K/AKT/mTOR and HIF1α),^[^ ^37^, ^38^ ^]^ CCT6A operates upstream by controlling inhibitory checkpoints rather than directly activating metabolic enzymes, revealing a novel layer of TME metabolic regulation.

Although β‐catenin can suppress the transcriptional activities of TXNIP and DDIT4, this regulation is not induced by direct promoter binding. Our mechanistic investigations revealed that CCT6A modulates β‐catenin phosphorylation dynamics: it reduces phosphorylation at Ser33/Ser37/Thr41 residues while enhancing phosphorylation at Ser552/Ser675 sites, thereby promoting β‐catenin nuclear translocation and facilitating its binding to the c‐Myc promoter. This phosphorylation switch ultimately drives transcriptional reprogramming and cisplatin resistance. Our findings expand the current understanding of Wnt/β‐catenin signaling, demonstrating that beyond the classical LEF/TCF7‐mediated transcriptional activation, this pathway can also orchestrate transcriptional repression through alternative transcription factor recruitment. However, our study still has certain limitations, and future work using phosphorylation site‐specific mutants will be necessary to validate the causal role of Ser552/Ser675 phosphorylation in mediating CCT6A function. Regarding other CAF‐derived factors, several secreted molecules such as IL‐6, SPP1, and TGF‐β have been shown to shape the TME and promote metabolic and immunosuppressive remodeling.^[^ ^39^, ^40^, ^41^ ^]^ Notably, while this study focuses on the cell‐autonomous functions of CCT6A in CAFs, we can not exclude the possibility that CCT6A functions in parallel or in synergy with these factors to reinforce the tumor‐promoting phenotype.

Another pivotal finding of our study is the elucidation of the molecular mechanism underlying the overexpression of CCT6A in GC. We first identified that CCT6P1, a pseudogene of CCT6A, is also highly expressed in GC. Although pseudogenes cannot encode functional proteins, they have emerged as crucial gene expression regulators through competitive microRNA (miRNA) binding. Functioning as molecular sponges, pseudogenes sequester miRNAs that would otherwise target their parental genes, thereby derepressing parental gene expression.^[^ ^42^ ^]^ Despite its lower expression level compared to CCT6A, CCT6P1 maintains substantial sequence homology that preserves shared miRNA binding sites. Through comprehensive miRNA target prediction analysis, we identified miR‐922 as a direct regulator of both CCT6P1 and CCT6A. Mechanistically, CCT6P1 binds and degrades miR‐922, thereby alleviating this post‐transcriptional repression mechanism and indirectly promoting CCT6A expression and GC progression.

Furthermore, we discovered a nuclear epigenetic mechanism involving c‐Myc‐mediated transcriptional activation of both CCT6A and CCT6P1. c‐Myc, which is aberrantly activated in over 50% of human cancers, serves as a master regulator of oncogenic processes including stemness maintenance,^[^ ^43^ ^]^ chemoresistance, and metabolic reprogramming.^[^ ^43^ ^]^ As a key driver of cancer, c‐Myc amplifies global transcription by promoting early transcription, especially the transcription of highly expressed genes. To enhance overall transcriptional output, c‐Myc modulates the residence time of transcriptional machinery components and controls the sequential entry and exit of different complexes to the promoter region.^[^ ^44^ ^]^ However, to determine which transcription complexes c‐Myc specifically recruits for the fine‐tuned regulation of CCT6P1 and CCT6A expression, further studies are needed to identify the complexes interacting with c‐Myc in specific contexts, such as in cells lacking CCT6P1 and CCT6A. While c‐Myc has been intensively studied as a therapeutic target,^[^ ^45^ ^]^ its direct pharmacological inhibition remains challenging. Intriguingly, our findings demonstrate that knockdown of either CCT6P1 or CCT6A significantly downregulates c‐Myc target genes in GC cells. Moreover, we established that CCT6A‐β‐catenin interaction enhances c‐Myc transcription, creating a self‐reinforcing oncogenic circuit through the c‐Myc/CCT6P1‐CCT6A axis. This positive feedback loop not only perpetuates malignant progression but also represents a promising therapeutic target for developing alternative c‐Myc‐directed therapies in GC.

## 4 Conclusion

We identified CAF‐derived exosomal CCT6A as a key driver of GC cell stemness, chemoresistance, and glycolysis. Mechanically, our study revealed that CCT6A plays a role in transcriptionally deactivating TXNIP and DDIT4 via β‐catenin/c‐Myc axis. Additionally, we revealed a crucial role for the pseudogene of CCT6A, CCT6P1, which functions as a ceRNA by sponging miR‐922 to stabilize CCT6A expression. Notably, c‐Myc directly upregulates both CCT6A and CCT6P1, forming a self‐sustaining positive feedback loop that amplifies oncogenic signaling and drives GC progression. This regulatory circuit, which includes CCT6A, CCT6P1, and the β‐catenin/c‐Myc axis, represents a synergistic mechanism that fosters GC malignancy. Our findings not only provide new insights into the molecular underpinnings of GC progression but also emphasize the pivotal role of CAF‐derived exosomal CCT6A in reprogramming the TME. Furthermore, the discovery of CCT6A as a modulator of chemoresistance and metabolic rewiring positions it as a promising biomarker for diagnosis and a potential therapeutic target in GC (Figure 8F). Future studies targeting the CCT6A/β‐catenin/c‐Myc axis may offer novel strategies to enhance the efficacy of GC treatment and improve patient outcomes.

## 5 Experimental Section

### Patients and Clinical Samples

A retrospective cohort enrolled 498 patients who underwent surgical resection and were diagnosed as GC from 2010 to 2012 at our hospital. In another retrospective cohort, 38 stage II‐III patients with GC who underwent surgical resection and postoperative therapy were also collected. Among which 19 cases recurrent after postoperative chemotherapy (RPC), and the other 19 cases with non‐recurrence (non‐RPC). Tissue samples were reviewed independently by two pathologists. None of the GC patients received preoperative therapy. All samples were obtained from the biobank of Fudan University Shanghai Cancer Center (FUSCC). The study was approved by the Research Ethics Committee, and all patients provided informed consent (Ethical code: 2407‐ZZK‐124).

### Isolation of Fibroblasts and Preparation of Conditioned Medium

CAFs and NFs were isolated from human GC and adjacent normal tissues obtained from patients who were undergoing surgery at the Department of Gastric Surgery, FUSCC. Tissues were first rinsed in phosphate‐buffered saline (PBS, Cat# abs962, Absin, Shanghai, China) supplemented with 100 U mL^−1^ penicillin and 100 µg mL^−1^ streptomycin (Cat# 15140122, Gibco, Gaithersburg, MD, USA), then minced into small fragments. Digestion was performed using 0.1% Type IV collagenase (Cat# C5138, Sigma‐Aldrich, Darmstadt, Germany) at 37 °C in a 5% CO_2_ incubator for 2 h. The reaction was halted by adding fetal bovine serum (FBS), and the cell suspension was centrifuged at 800 × *g* for 5 min. The resulting cell pellet was resuspended and transferred to a new culture dish. CAFs and NFs were purified after two passages and subsequently used for functional assays.

Fibroblasts were maintained in RPMI‐1640 medium (Cat# C22400500BT, Gibco, Gaithersburg, MD, USA) supplemented with 10% FBS (Cat# 10270‐106, Gibco, Gaithersburg, MD, USA) until reaching 70–90% confluence. The culture medium was then removed, and fresh serum‐free RPMI‐1640 medium was added. After 48 h of incubation, the conditioned medium was collected, centrifuged at 1000 × *g* for 5 min, and designated as CAF‐CM

### CAF‐Cancer Cell Co‐Culture System

HGC‐27 or MKN‐45 cells (1 × 10^5^) were seeded at the bottom of the six‐well plate (Cat# 3516, Corning, NY, USA), while CAFs (5 × 10^4^) were incubated in the tissue culture plate insert (Cat# 14111, Labselect, Hefei, China), which is the upper chamber of a 6‐well Transwell apparatus. The insert contained a membrane of 0.4 µm pore size, allowing the exchange of supernatants but not cells. After 1 week of co‐culture, cells were collected for further experiments.

### Exosome Isolation and Labeling

Exo were isolated from the conditioned medium of CAFs by differential ultracentrifugation. Cells were grown to 80% confluence in T175 flasks, after which the culture medium was replaced for Exo‐collection medium (culture medium without FCS and Primocin). After 24 h, the conditioned medium was harvested and centrifuged for 15 min at 1500×g to remove cellular debris, after which larger microvesicles were removed by centrifugation for 30 min at 10 000×g. Exo were isolated by centrifugation for 60 min at 100 000×g, and subsequently washed twice by re‐suspension in PBS and centrifugation for 60 min at 100 000×g, after which the final Exo pellet was collected by centrifugation at 100 000×g.

Exo were labeled with the red fluorescent dye PKH67 (Sigma). The exo were resuspended in 180 µL PBS, and 20 µL of Diluent C containing PKH67 (diluted 1:25) was added. The mixture was gently pipetted for 30 s and incubated at RT for 5 min. To terminate the reaction, 500 µL of 10% BSA in PBS was added. Next, serum‐free media was added to adjust the total volume to 3.5 mL. The Exo suspension was then placed on top of a 0.971 m sucrose (in PBS) layer and centrifuged at 190.000 × g for 2 h to pellet Exos, while unbound dye remained on top of the sucrose layer. The pelleted Exo were resuspended in PBS, centrifuged at 100 000 × *g*, and subsequently resuspended in the appropriate buffer or medium for further analysis.

### Identification of Exosomes

The exosomes were diluted in PBS at 1:10 ratio, and 15 µL of solution was added to a carbon‐coated grid. The grid was allowed to sit statically for 1 min, then excess solution was absorbed. The samples were stained with 2 % uranyl acetate for 10 min, dried, and visualized under an electron microscope. The concentration and size of the exosomes were assessed using a nanoparticle analyzer (NTA), with deionized water used as the reference. The assessment was based on the principle of Brownian motion. Western blotting was performed to detect exosome‐specific marker proteins, CD81 and CD63, to confirm the identity of the exosome. Details of the antibodies used are provided in Table S4 (Supporting Information).

### Immunofluorescence for Cells

Cells were cultured on coverslips and fixed using 4% paraformaldehyde at room temperature for 15 min. After fixation, the cells were permeabilization with 0.1% Triton X‐100 for 10 min. Nonspecific binding was blocked by incubating the cells for 1 h with 5% bovine serum albumin (BSA). The primary antibodies were applied and incubated overnight at 4 °C. Following washing, fluorophore‐conjugated secondary antibodies were added and introduced at RT for 1 h. Nuclei were stained with DAPI for 5 min. Finally, the samples were mounted with anti‐fade medium and examined under a fluorescence microscope. Images were captured for analysis of protein localization and expression. Details of the antibodies used are provided in Table S4 (Supporting Information).

### Fluorescence In Situ Hybridization (FISH) and Fluorescent Multiplex Immunohistochemistry (mIHC), Tissue Imaging, and Analysis

FISH for CCT6P1 was performed using the multiplex fluorescence RNA in situ hybridization kit (Alpha X Bio, Beijing, China). Cells were fixed with 10% NBF and incubated with preA solution at RT to inhibit endogenous peroxidase activity. After proteinase treatment, the samples were hybridized with probes at 40 °C for 2 h, followed by signal amplification. The target RNA was labeled with green fluorescence using tyramide signal amplification (TSA) and a tyramide substrate. Formalin‐fixed paraffin‐embedded (FFPE) tissue sections (2–5 µm) were deparaffinized with xylene, rehydrated through a gradient of ethanol, and subjected to heat‐induced epitope retrieval (HIER) using EDTA buffer. IHC staining was conducted as described in a previous study.^[^ ^20^ ^]^ The images were separately overlaid and analyzed using HALO software. Antibody details are provided in Table S4 (Supporting Information).

### Western Blot Analysis

Total protein was extracted from cells and tissues and subjected to sodium dodecyl sulfate‐polyacrylamide gel electrophoresis (SDS‐PAGE). Proteins were then transferred onto polyvinylidene difluoride (PVDF) membranes, blocked, and incubated with primary antibodies at 4 °C overnight. After three washes with TBS‐T (10 min each), membranes were incubated with goat anti‐rabbit IgG secondary antibody (1:1000) at 37 °C for 1 h. Protein signals were detected using an enhanced chemiluminescence (ECL) kit (No. 35055, Thermo Scientific). Antibody details are provided in Table S4 (Supporting Information).

### Quantitative Reverse Transcription Polymerase Chain Reaction (RT‐qPCR)

Total RNA was extracted using the RNeasy Kit (AM1924, Invitrogen) according to the manufacturer's instructions. Reverse transcription was performed to generate cDNA, which was then used for target gene amplification. Glyceraldehyde 3‐phosphate dehydrogenase (GAPDH) served as an internal control. Primer sequences used for RT‐qPCR analysis are provided in Table S5 (Supporting Information).

### Plasmid Construction and Viral Infection

To generate stable cell lines, lentiviruses (Transheep, China) carrying scrambled shRNA (shNC) or specific shRNAs targeting signal transducer and CCT6A (shCCT6A) were transduced into CAF cells. Lentiviruses were generated by HEK293T cells with recombinant vectors and the pPACK Packaging Plasmid Mix (Transheep). shRNA vectors were constructed by cloning RNA fragments into pSIH‐H1‐Puro (SBI), and overexpression vectors were generated by inserting amplified gene fragments into pCDH (Transheep).

### Transfection

For transfection, cells were starved 2 h prior to transfection, following the manufacturer's protocols using Lipofectamine 3000 (L3000015, Invitrogen). At 40 h post‐transfection, cells were treated with complete media for the indicated period before harvesting for further analysis. Details of the transfection products used are shown in Table S6 (Supporting Information).

### RNA Sequencing

Total RNA was extracted from cells using TRNzol (Cat# DP424, Tiangen, Beijing, China). Quality control, library preparation, RNA sequencing analysis, and KEGG enrichment analysis were carried out by NOVELBIO. Differentially expressed genes were identified with an adjusted *p* value ≤0.05, log2 FC>1.

### Immunoprecipitation and Protein Mass Spectrometry Assay

Immunoprecipitation (IP) and protein mass spectrometry (MS) assays were performed as previously described.^[^ ^45^, ^46^ ^]^ Protein A/G agarose (Santa Cruz Biotechnology, USA) was used for IP. Total proteins were extracted from the GC cell lines using IP lysis buffer, precleared with microspheres, and incubated with anti‐CCT6A (Cat# 19793‐1‐AP, Proteintech) at 4 °C overnight. The following day, the immune complex samples were incubated with protein A magnetic beads at room temperature for 20 min, and the bound proteins were collected by centrifugation. Finally, the retrieved proteins were detected by Western blot analysis or resolved by in‐gradient gel electrophoresis, followed by MS identification.

### Chromatin Immunoprecipitation and Luciferase Reporter Assays

A chromatin immunoprecipitation (ChIP) assay kit (Upstate, Billerica, MA) was used according to the manufacturer instructions. Briefly, cells were fixed with formaldehyde, and DNA was sheared to fragments at 200–1000 bp by repeated sonication. Chromatin was then incubated and precipitated with antibodies against c‐Myc or IgG. Luciferase reporter assay was performed using a Dual Luciferase Assay Kit (Promega, Madison, WI, USA). The wild type (WT) and mutant target genes were inserted into the pGL3 plasmid. Transient transfection was performed using Lipofectamine 3000 (Thermo Fisher, Carlsbad, CA, USA) according to the manufacturer's instructions. One or 2 days later, luciferase activity was measured with a Dual‐Glo Luciferase Assay System (Promega), and Renilla luciferase activity was used to normalize the firefly luciferase activity.

### Apoptosis Assay

HGC‐27 and MKN‐45 cells were treated with cisplatin or vehicle and then digested by 0.25% trypsin. After centrifugation, apoptosis was detected using the Annexin V‐FITC/PI Apoptosis Detection Kit (Cat# 40302ES60, Yeasen, Shanghai, China). Cells were incubated with 5 µL of Annexin V‐FITC and 10 µL PI Staining Solution for 15 min, and apoptosis was analyzed immediately using a flow cytometer (#CytoFlex, Beckman, Brea, CA, USA). Annexin V+/PI‐ indicates early apoptosis, while Annexin V+/PI+ indicates late apoptosis.

### Enzyme‐Linked Immunosorbent Assay (ELISA)

Exosomes from cell culture supernatants were isolated by differential ultracentrifugation. Briefly, supernatants were sequentially centrifuged at 1500×*g* for 15 min, to remove cellular debris, after which larger microvesicles were removed by centrifugation for 30 min at 10 000×*g*. Exo were isolated by centrifugation for 60 min at 100 000×*g*, and subsequently washed twice by re‐suspension in PBS and centrifugation for 60 min at 100 000×*g*, after which the final Exo pellet was collected by centrifugation at 100 000 × *g* to pellet exosomes. The exosome pellet was washed in PBS and subjected to an additional centrifugation step. Detection of exosome and supernatant CCT6A protein levels using ELISA(Cat# JY2127, Aiyou Biotechnology Center, Shanghai, China). For serum samples, due to limited volume per patient (≈300 µL), exosome isolation was not feasible. Thus, total CCT6A levels were measured directly from whole serum.

### Glucose, Lactate, and ATP Assay

Cells were plated into a 12‐well plate and incubated in RPMI‐1640 containing 10% FBS for 10 h. To measure the glucose uptake, lactate secretion, and ATP generation in the cell supernatant, the following kits were used according to the manufacturer's instructions: Glucose uptake kits (BioVisoin, K676‐100), Lactate detection kits (BioVisoin, K627‐100), and ATP generation kits (Beyotime, S0027).

### Oxygen Consumption Rate and Glycolytic Proton Efflux Rate

The glycolytic capacity was determined using the Glycolysis Stress Test Kit, following the manufacturer's instructions. Briefly, 4 × 10^4^ cells were seeded into 96‐well plates and incubated overnight. The culture medium was then replaced with XF basic culture medium before measuring the oxygen consumption rate (OCR). After adding 2 um oligomycin, 1.5 um carbonyl cyanide 4‐(trifluoromethoxy) phenylhydrazone (FCCP), 2 um rotenone, and 2 um antimycin A, the OCR was determined by a Seahorse XF24 Extracellular Flux Analyzer (Seahorse Bioscience, USA). For the glycolytic rate assay, cells were plated in the Seahorse XF Glycolytic Rate Assay Medium containing glucose (5 mm) and HEPES buffer (10 mm), and the basal rates were recorded over three measurement periods. Subsequently, AA/Rot were injected. The glycolytic proton efflux rate (PER) was calculated by subtracting the mitochondrial acidification from the total proton efflux rate (PER). The OCR and GlycoPER values were normalized to cell number and are presented as the mean ± SD.

### Organoid Isolation and Culture

Freshly collected tissues were immediately placed in tissue storage solution. GC tissues were digested, filtered through a 70 µm filter, and centrifuged at 300×g. After enzymatic digestion (trypsin, collagenase IV, DNase I) at 37 °C for 30–60 min, cell aggregates were filtered (70 µm), mixed with Matrigel (1:1), and seeded in 24‐well plates. After polymerization (37 °C, 15 min), organoid culture medium was added, and cultures were maintained at 37 °C, 5% CO_2_. Organoids were harvested after 7–14 days for passaging and characterized by H&E staining. For viral transfection, organoids were incubated with viral suspension (4 °C, 1 h), infected (6–8 h), and resuspended in Matrigel for further culture. For drug assays, organoids in 96‐well plates were treated with etoposide, and viability was assessed using Cell Titer‐Glo 3D (Nanjing Vazyme Biotech) to evaluate proliferation inhibition and apoptosis induction.

### In Vivo Experiments

BALB/c‐nude mice (aged 5 weeks) were maintained under pathogen‐free conditions. MKN‐45 cell lines with CAFs (with or without CCT6A knockdown) at a 1:3 ratio in serum‐free medium were injected subcutaneously into flanks of the nude mice. The length (L) and width (W) of the tumors were measured starting on the seventh day. Tumor volume was calculated with the formula: V = (L×W^2^)/2. Mice were intraperitoneally injected with β‐catenin inhibitor XAV939 (20 mg kg^−1^), MYC blocker 10058‐F4 (15 mg kg^−1^) or PBS every other day for a total of 8 injections. After 21 days, all mice were sacrificed, and the tumors were excised and measured. Tumor volumes were calculated with the following formula: V = 1/2 × long diameter × square of short diameter. The animal studies were reviewed and approved by the Ethics Committee for Animal Studies of Shanghai University (SYZ201809003).

### Statistical Analysis

All experiments were independently performed at least in triplicate, and data are presented as mean ± standard deviation (SD). For statistical analysis, the Mann–Whitney U test was used to compare two unpaired, non‐normally distributed groups, including cell population proportions, quantification of immunohistochemistry staining, and relative gene expression data. Student's t‐test was applied to normally distributed two‐group comparisons, and one‐way ANOVA was used for multiple group comparisons with normal distribution. Kaplan–Meier survival analysis with log‐rank test was used to assess differences in overall and progression‐free survival. Univariate and multivariate Cox proportional hazards models were employed to identify independent prognostic factors. All statistical analyses were conducted using GraphPad Prism 9.0. Statistical significance was defined as ^*^ *p *< 0.05, ^**^ *p *< 0.01, ^***^ *p *< 0.001, ^****^ *p *< 0.0001, while “ns” indicates no statistically significant difference.

## Conflict of Interest

The authors declared no conflict of interest.

## Author Contributions

H.S., T.Q.Z., X.Y.Z., and Y.X.L. contributed equally to this work. M.D.X., H.S., and T.Q.Z. conceived the study, performed the literature search and bioinformatics analysis, and prepared the figures. H.S. performed the in vitro and in vivo experiments. W.F.W, T.Q.Z, X.Y.Z, Y.X.L, X.W, X.W, C.T, S.J.N, W.W.W, M.Z, D.H, L.W, X.Y.W., and X.W. helped with data collection, analysis, and interpretation. W.F.W., M.D.X., and W.Q.S. wrote and revised the manuscript. All authors read and approved the final manuscript.

## Supporting information



Supporting Information

## Data Availability

The data that support the findings of this study are available from the corresponding author upon reasonable request.
