# Supplementary material for: Exosomal CCT6A Secreted by Cancer‐Associated Fibroblasts Interacts with β‐Catenin to Enhance Chemoresistance and Tumorigenesis in Gastric Cancer
Source: Adv Sci (Weinh). 2025 Aug 13;12(38):e06674. doi: 10.1002/advs.202506674 (PMC12520511; doi:10.1002/advs.202506674)
Supplement: Supplementary file 1 — Supporting Information [file ADVS-12-e06674-s001.docx]

**Supplementary Figure Legend**

**Figure S1.** **The transcriptional targets of c-Myc, and the positive correlation of CCT6P1 and CCT6A.**


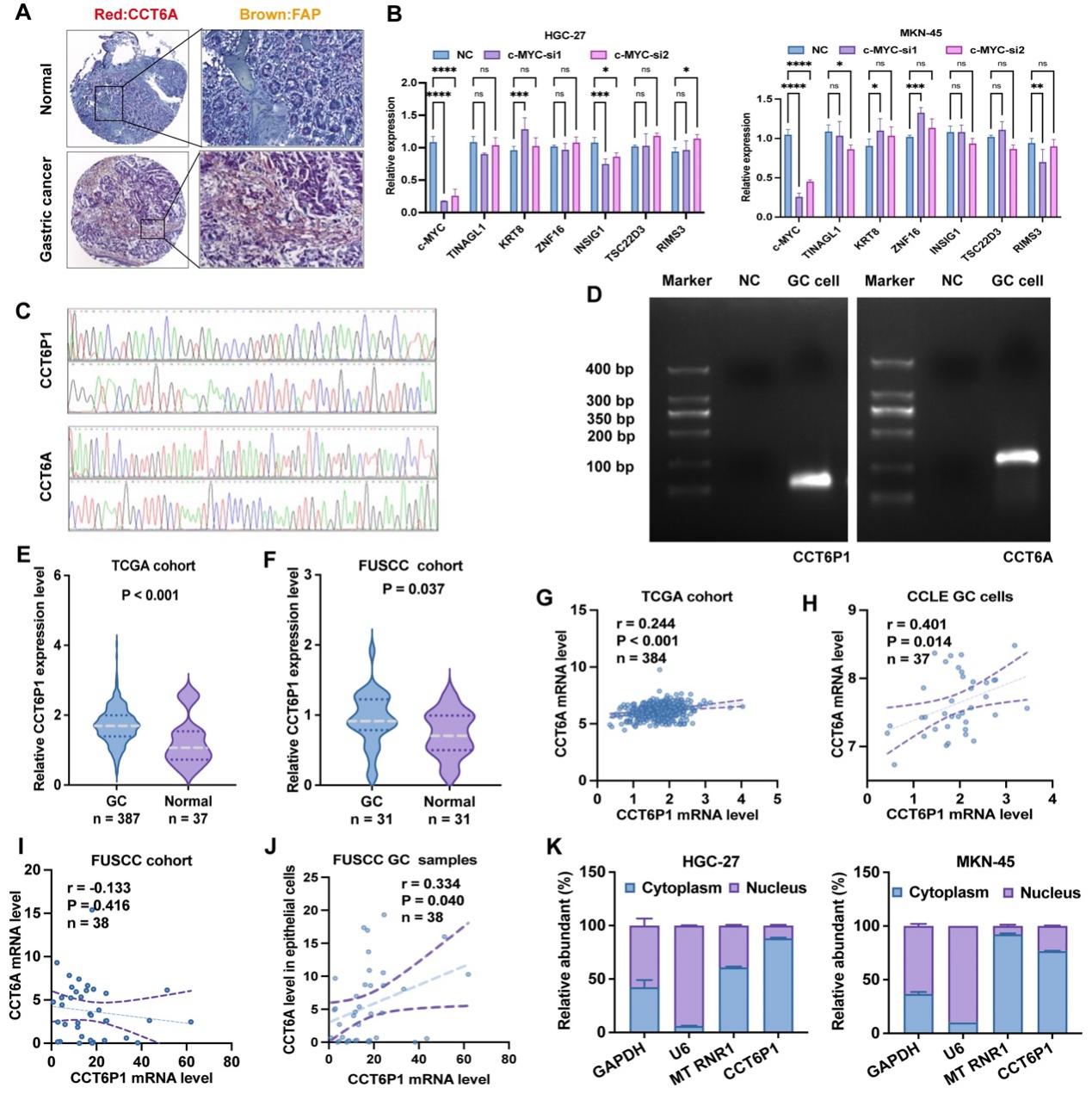


1. Dual-immunohistochemical (IHC) staining of CCT6A and FAP in matched adjacent normal and GC tissues.
2. RT-qPCR quantification of TINAGL1, KRTA8, ZNF16, INSIG1, TSC22D3, and RIMS3 mRNA levels in c-Myc-knockdown HGC-27 and MKN-45 cells.

C-D. RNA sequencing (C) and DNA gel (D) analysis of CCT6A and CCT6P1.

E-F. Box plots showing elevated CCT6P1 mRNA levels in GC tissues compared to normal tissues from the TCGA cohort (E) and FUSCC cohort (F).

G-J. Pearson correlation analysis of CCT6A and CCT6P1 expression in the TCGA cohort (G), the CCLE cohort (H) and the FUSCC cohort (I-J).

K.  Subcellular fractionation RT-qPCR of CCT6P1 RNA localization.

(Data represent mean ± SEM. p-values are determined by one-way ANOVA，Mann Whitney U-test, Pearson r and Chi square test. *ns, not significant; *P＜0.05; **P＜0.01; ***P＜0.001; ****P＜0.0001)*.

**Figure S2. CCT6A is a ceRNA target of the CCT6P1/miR-922 interaction.**


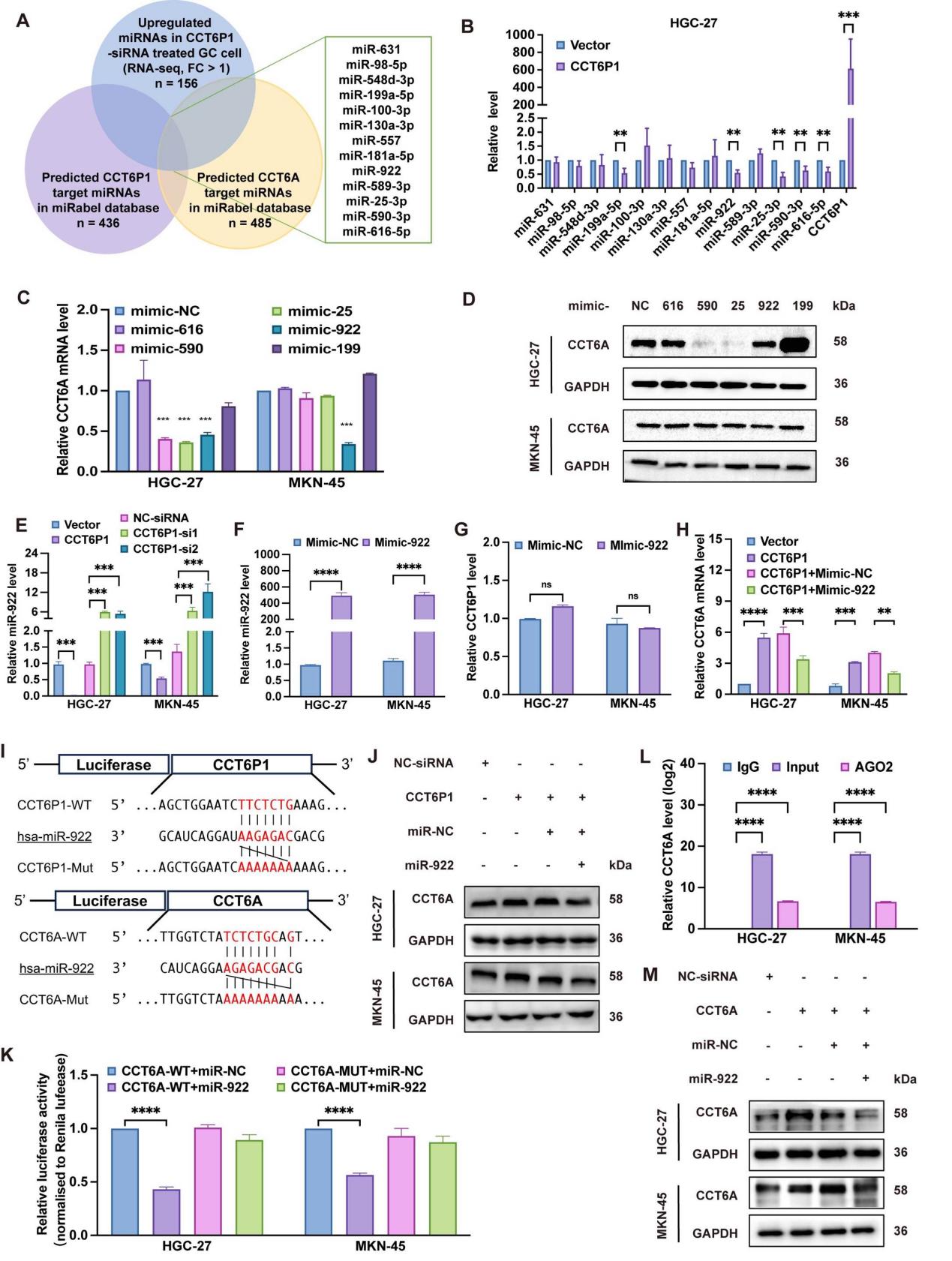


A. miRNA target prediction (miRabel) and RNA-seq intersection analysis identifying 13 miRNAs with shared 3′UTR binding sites in both CCT6A and CCT6P1.

B. RT-qPCR analysis of the expression levels of 13 miRNA levels in HGC-27 and MKN-45 cells with CCT6P1 overexpression.

C-D. RT-qPCR and Western blot analysis of mRNA and protein levels of CCT6A in HGC-27 and MKN-45 cells transfected with miR-616, miR-590-3p, miR-25, miR-922 and miR-199a mimics.

E. RT-qPCR analysis of miR-922 expression levels in HGC-27 and MKN-45 cells with CCT6P1 overexpression or knockdown.

F-G. RT-qPCR analysis of CCT6P1 and CCT6A expression levels in HGC-27 and MKN-45 cells with miR-922 mimic.

H．RT-qPCR analysis of CCT6A expression levels in HGC-27 and MKN-45 cells with CCT6P1 or co-transfection with miR-922 mimic.

I. Schematic diagram showing the putative miR-922 binding sites in CCR6A and CCT6P1.

J. Western blot analysis of CCT6A expression levels in HGC-27 and MKN-45 cells with CCT6P1 overexpression or co-transfection with miR-922 mimic.

K. Relative luciferase activities of wild type (WT) and mutated (Mut) CCT6A reporter plasmid in HGC-27 and MKN-45 cells, or co-transfected with miR-922 mimic.

L. Anti-Ago2 RIP was used to pull down endogenous RNAs associated with Ago2, IgG was served as the control. The levels of CCT6A were measured by RT-qPCR, and the data are presented as fold enrichment in Ago2 relative to input.

M. Western blot analysis of CCT6A expression levels in HGC-27 and MKN-45 cells with CCT6A overexpression of co-transfection with miR-922 mimic.

(Data represent mean ± SEM. p-values are determined by Mann Whitney U-test and one-way ANOVA. *ns, not significant; *P＜0.05; **P＜0.01; ***P＜0.001; ****P＜0.0001* ).

**Figure S3.** **CAF-derived CCT6A transport to GC cells via extracellular vesicles.**

**
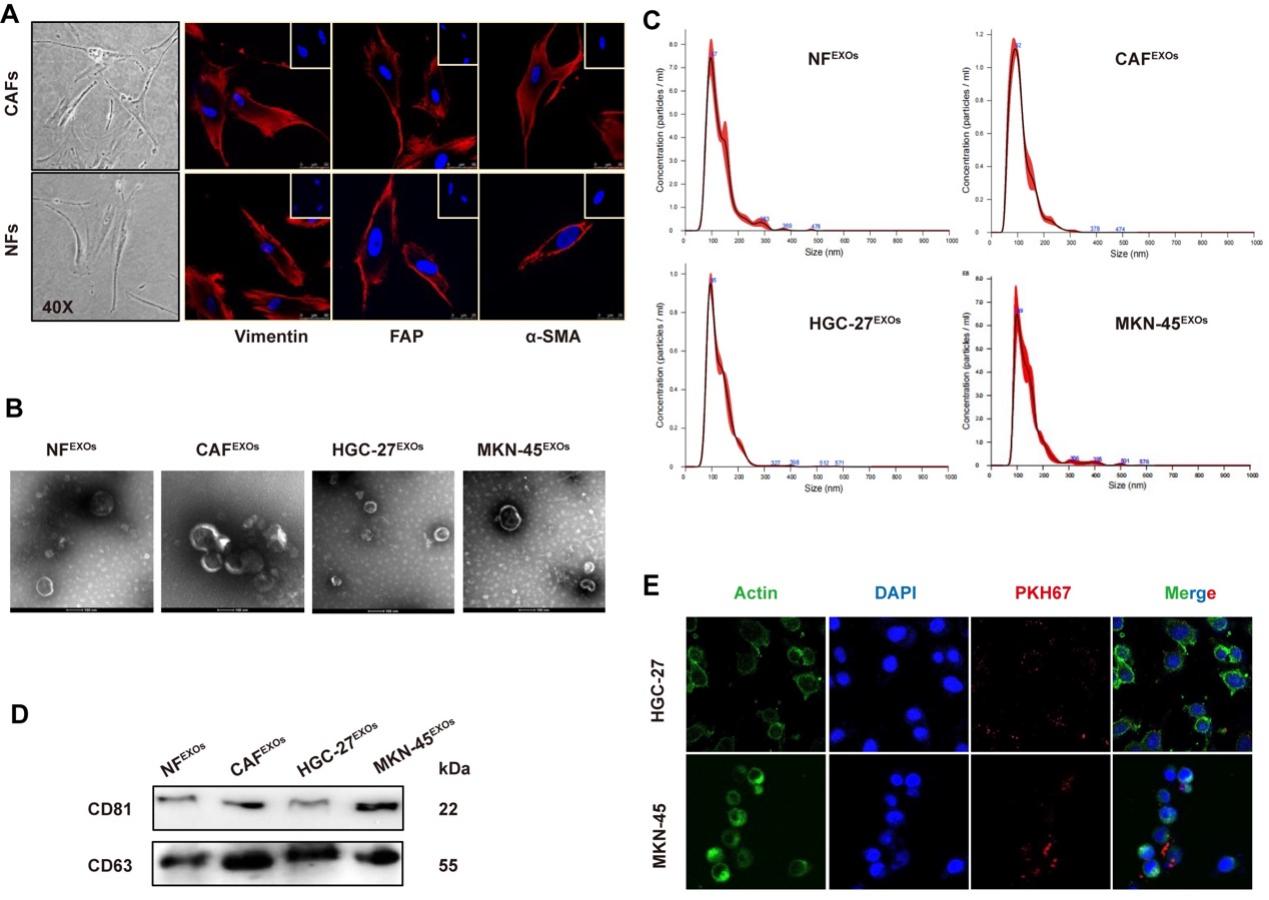
**

A. Immunofluorescent staining showing the expression Vimention, FAP and α-SMA

expression in CAFs and NFs (scale bar = 25 µm).

B-D. Transmission electron microscopy, nanoparticle tracking analysis, and Western blot confirm the extraction of extracellular vesicles.

E. Immunofluorescent staining showing that CAF-derived exosomes can be uptake by HGC-27 and MKN-45 cells.

**Figure S4.** **CAFs-derived exosomal CCT6A enhances glycolysis in GC by activating β-catenin/DDIT4-TXNIP axis.**

**
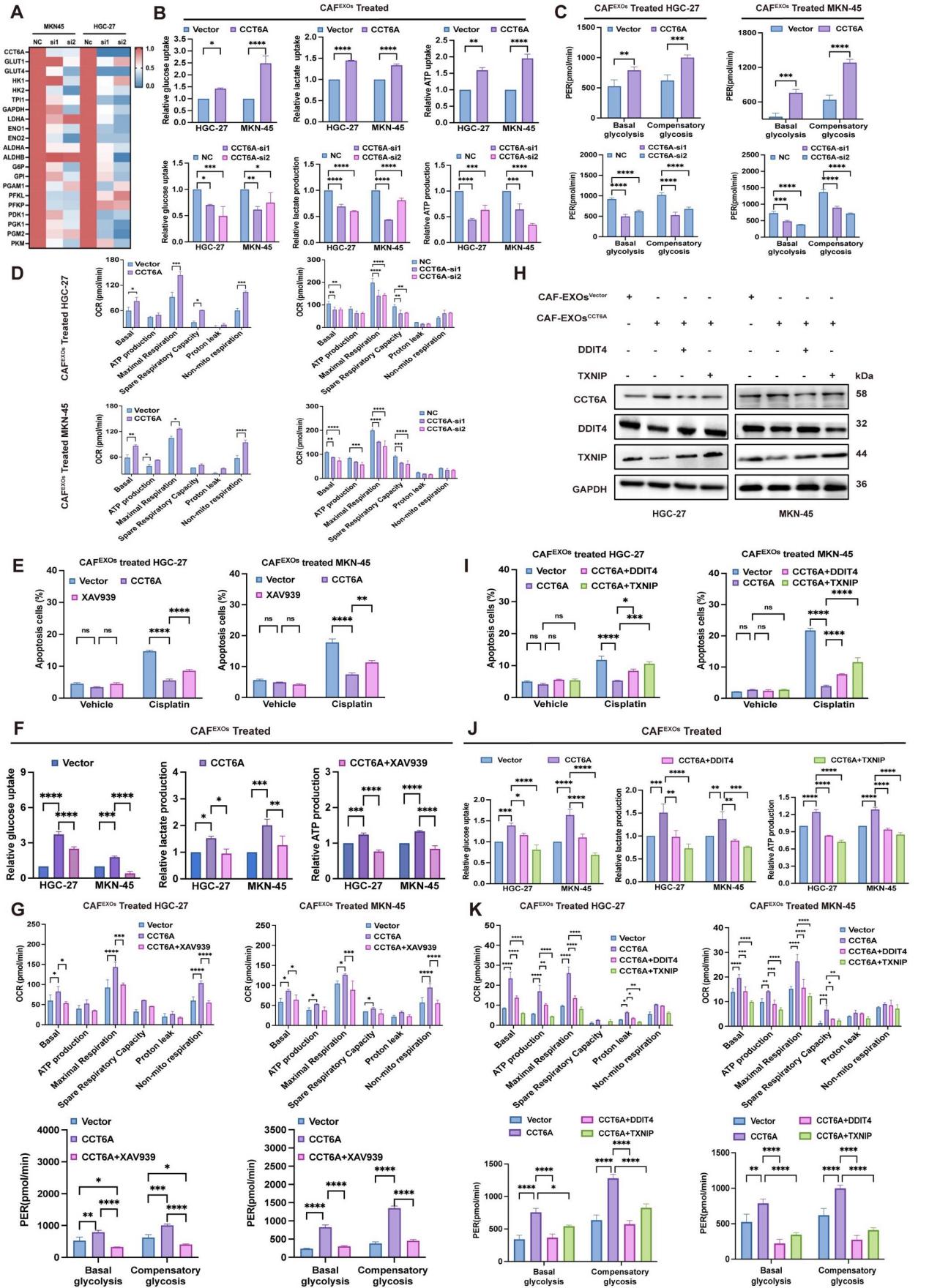
**

A. Heatmap representing significantly dysregulated genes from RT-qPCR analysis of rate-limiting glycolytic enzymes in HGC-27 and MKN-45 cells treated with CCT6A knockdown.

B. Quantitative results of glucose uptake, ATP production, lactate production in HGC-27 and MKN-45 cells treated with CCT6A knockdown or overexpression *via* CAF-derived exosomes.

C-D. Quantitative results of seahorse extracellular flux analysis measured glycolytic proton efflux rate (GlycoPER) and oxygen consumption rate (OCR) in HGC-27 and MKN-45 cells treated with CCT6A knockdown or overexpression *via* CAF-derived exosomes or CAFs under cisplatin -treated.

E. Quantitative results of flow cytometry analysis of apoptotic cells in HGC-27 and MKN-45 cells treated with CCT6A vector CAF EXOs, CCT6A overexpression CAF EXOs, CCT6A overexpression CAF EXOs+XAV939.

F-G. Quantitative results of glucose uptake, ATP production, lactate production, GlycoPER and OCR in HGC-27 and MKN-45 cells treated with CCT6A vector CAF EXOs, CCT6A overexpression CAF EXOs, CCT6A overexpression CAF EXOs with XAV939.

H. Western blot analysis of CCT6A, TXNIP, and DDIT4 levels in HGC-27 and MKN-45 cells with CCT6A or co-transfection with DDIT4 and TXNIP.

I. Quantitative results of flow cytometry analysis of apoptotic cells in HGC-27 and MKN-45 cells treated with with CCT6A or co-transfection with DDIT4 and TXNIP.

J-K. Quantitative results of glucose uptake, ATP production, lactate production, glycolytic proton efflux rate (GlycoPER) and oxygen consumption rate (OCR) in HGC-27 and MKN-45 cells treated with CCT6A or co-transfection with DDIT4 and TXNIP.

(Data represent mean ± SEM. p-values are determined by Mann Whitney U-test and one-way ANOVA. *ns, not significant; *P＜0.05; **P＜0.01; ***P＜0.001; ****P＜0.0001*)*.*

**Supplementary Table S1.** Relationship between CCT6A expression in the gastric cancer stroma and clinicopathological parameters of gastric cancer patients.

| Variables | CCT6A expression in CAFs | | P value |  |
| --- | --- | --- | --- | --- |
|  | Low (n=185) | High (n=287) |  |  |
| **Age**  <60  ≥60 |  |  |  |  |
|  | 4（2.2）  181（97.8） | 5（1.7）  282（98.3） | 1 |  |
| **Gender** |  |  |  |  |
| Male | 128（69.2） | 73（25.4） | 0.242 |  |
| Female  **Differentiation** | 57（30.8） | 214（76.4） |  |  |
|  |  |  |  |  |
| Well/ moderated  Poor/Undifferentiated | 16 (8.6)  169 (91.4) | \| 35 (12.4) \| \| --- \| \| 248 (87.6) \| | 0.267 |  |
| **Vascular invasion**  Absent  Present | 102 (55.1)  83 (44.9) | 121 (42.2)  166 (57.8) | **0.008*** |  |
| **Nervous invasion**  Absent  Present | 94 (50.8)  91 (49.2) | \| 129 (44.9) \| \| --- \| \| 158 (55.1) \| | 0.250 |  |
| **Lauren**  Intestinal type  Diffuse type  Mixed type  Unknown | 60 (35.3)  26 (15.3)  74 (43.5)  10 (5.9) | 106 (51.5)  33 (16.0)  59 (28.6)  8 (3.9) | **0.007*** |  |
| **T stage**  T1  T2  T3  T4 | 26 (14.3)  20 (11.0)  1 (0.5)  135 (74.2) | 17 (5.9)  34 (11.9)  12 (4.2)  223 (78.0) | **0.003*** |  |
| **N stage**  N0  N1  N2  N3 | 61 (33.0)  47 (25.4)  38 (20.5)  39 (21.1) | 73 (25.4)  103 (35.9)  45 (15.7)  66 (23.0) | **0.049*** |  |
| **M stage**  M0 | 181(97.8) | 259 (90.2) | **0.003*** |  |
| M1 | 4(2.2) | 28(9.8) |  |  |
| **TNM stage**  I  II  III  IV | 34 (18.4)  50 (27.0)  97 (52.4)  4 (2.2) | 29 (10.1)  74 (25.8)  157 (54.7)  27 (9.4) | **0.002*** |  |

**Supplementary Table S2．**Univariate and multivariate analyses of clinicopathological factors for overall survival in gastric cancer patients.

| Variable | | Univariate analysis | Multivariate analysis | | |
| --- | --- | --- | --- | --- | --- |
|  |  | HR (95 % CI) | p^a^ | HR (95 % CI) | p^a^ |
| Age  (≥ 60/<60) | | 1.49(1.03-2.16) | **0.036** | 1.47(1.00-1.65) | **0.036** |
| Gender  (Male/Female) | | 0.91(0.61-1.37) | 0.650 |  |  |
| Location  (Cardia/Fundus/  Antrum/Other) | | 0.96(0.78-1.80) | 0.704 |  |  |
| Tumor mass size  (≥ 5/< 5) | 1.16(0.80-1.68) | | 0.426 |  |  |
| Histologic grade  (Poor, Undifferented/  Good, mod) | 1.40(0.68-2.88) | | 0.356 |  |  |
| Vascular invasion  (Present/Absent) | 1.88(1.29-2.74) | | **<0.001** |  |  |
| Nervous invasion  (Present/Absent) | 2.04(1.39-3.00) | | **<0.001** |  |  |
| N stage (quarter method)  (Present/Absent) | 1.60(1.36-1.89) | | **<0.000** |  |  |
| Distant metastasis  (Present/Absent) | 8.13(3.90-16.96) | | **<0.000** | 3.71(1.45-9.49) | **0.006** |
| pTNM stage  (III+IV/I+II) | 2.87(2.04-4.03) | | **<0.000** | 1.67(1.03-2.70) | **0.036** |
| FAP protein  (High/Low) | 1.35(0.89-2.05) | | 0.152 |  |  |
| CCT6A protein  (High/Low) | 1.39(0.93-2.09) | | 0.109 |  |  |
| CCT6A&FAP protein  (High/Low) | 1.55(1.07-2.26) | | **0.0217** | 1.60（1.09-2.36） | **0.016** |
| DDIT4 protein  (High/Low) | 0.57(0.37-0.88) | | **0.011** | 0.80（0.53-1.23） | **0.040** |
| TXNIP protein  (High/Low) | 0.66(0.45-0.99) | | **0.043** |  |  |

**Supplementary Table S3.** Univariate and multivariate analyses of clinicopathological factors for disease free survival in gastric cancer patients.

| Variable | Univariate analysis | | | Multivariate analysis | |
| --- | --- | --- | --- | --- | --- |
|  | HR (95 % CI) | p^a^ | HR (95 % CI) | | p^a^ |
| Age  (≥ 60/< 60) | 1.35(0.94-1.94) | 0.102 |  | |  |
| Gender  (Male/Female) | 1.02(0.69-1.50) | 0.939 |  | |  |
| Location  (Cardia/Fundus/  Antrum/Other) | 0.94 (0.77-1.15) | 0.573 |  | |  |
| Tumor mass size  (≥ 5/< 5) | 1.19(0.83-1.70) | 0.354 |  | |  |
| Histologic grade  (Poor, Undifferented/  Good, mod) | 1.28(0.65-2.53) | 0.471 |  | |  |
| Vascular invasion  (Present/Absent) | 1.83(1.27-2.63) | **<0.001** |  | |  |
| Nervous invasion  (Present/Absent) | 2.09(1.43-3.06) | **<0.001** |  | |  |
| N stage (quarter method）  (Present/Absent) | 1.59(1.35-1.87) | **<0.000** |  | |  |
| Distant metastasis  (Present/Absent) | 9.37(4.48-19.62) | **<0.000** | 3.62(1.43-9.17) | | **0.007** |
| pTNM stage  (III+IV/I+II) | 2.87(2.05-4.01) | **<0.000** | 1.71(1.07-2.73) | | **0.025** |
| FAP protein  (High/Low) | 1.34(0.90-2.01) | 0.154 |  | |  |
| CCT6A protein  (High/Low) | 0.081(0.96-2.13) | 0.539 |  | |  |
| CCT6A&FAP protein (High/Low) | 1.56 (1.08-2.26) | **0.017** | 1.53(1.05-2.24) | | **0.026** |
| DDIT4 protein  (High/Low) | 0.57(0.37-0.87) | **<0.009** | 0.58 (0.37-0.91) | | **0.018** |
| TXNIP protein  (High/Low) | 0.78(0.54-1.14) | 0.203 |  | |  |

**Supplementary Table S4.** Antibodies used in this study.

| Antibodies | Epitope | SOURCE | IDENTIFIER | Experiment |  |  |
| --- | --- | --- | --- | --- | --- | --- |
| Anti-CD81[D3N2D] | Rabbit monoclonal | CST | CST56039 | WB |  |  |
| Anti-CD63[E1W3T] | Rabbit monoclonal | CST | CST52090 | WB |  |  |
| Anti-AXIN1[C76H11] | Rabbit monoclonal | CST | CST2087 | WB |  |  |
| Anti-DVL1[30D2] | Rabbit monoclonal | CST | CST3224 | WB |  |  |
| Anti-GSK-3β[D5C5Z] | Rabbit monoclonal | CST | CST12456 | WB |  |  |
| Anti-DDDDK tag  [EPR20018-251] | Rabbit monoclonal | Abcam | ab205606 | WB |  |  |
| Anti-β-Catenin [D10A8] | Rabbit monoclonal | CST | CST8480 | WB |  |  |
| Anti-Phospho-β-Catenin (Ser33/37/Thr41) | Rabbit monoclonal | CST | CST9561 | WB |  |  |
| Anti-Phospho-β-Catenin(Ser552) | Rabbit monoclonal | CST | CST9566 | WB |  |  |
| Anti-Phospho-β-Catenin (Ser675) [D2F1] | Rabbit monoclonal | CST | CST4176 | WB |  |  |
| Anti-Phospho-β-Catenin(Ser45) | Rabbit monoclonal | CST | CST9564 | WB |  |  |
| Anti-GAPDH[6C5] | Mouse monoclonal | Abcam | ab8245 | WB |  |  |
| Anti-beta Actin | Mouse monoclonal | Abcam | ab8226 | WB |  |  |
| Anti-CD133 | Rabbit polyclonal | Proteintech | 18470-1-AP | WB |  |  |
| An-SOX4 | Rabbit polyclonal | Proteintech | 27414-1-AP | WB |  |  |
| Anti-CCT6A | Rabbit polyclonal | Proteintech | 19793-1-AP | IP and WB |  |  |
| Anti-CCT6A | Rabbit polyclonal | Sigma | HPA042996 | mIHC |  |  |
| Anti-FAP[EPR20021] | Rabbit polyclonal | Abcam | ab207178 | WB, mIHC and IF |  |  |
| Anti-pan-CK[C-11] | Mouse monoclonal | Abcam | ab7753 | mIHC and WB |  |  |
| Anti-TXNIP | Rabbit polyclonal | Bioworld | BS65874 | mIHC and WB |  |  |
| Anti-DDIT4 | Rabbit polyclonal | Sigma | HPA034508 | mIHC and WB |  |  |
| Anti-α-SMA[D4K9N] | Rabbit monoclonal | Abcam | ab207178 | WB and IF |  |  |
| Anti-Vimentin[D21H3] | Rabbit monoclonal | CST | CST5741 | IF |  |  |
| An-Ki-67[8D5] | Rabbit monoclonal | CST | CST9499 | IHC |  | |

**Supplementary Table S5.** The primer sequences used in this study.

| CCT6A-F | 5’-GGCACCATGAAGATGCTCGT-3’ |
| --- | --- |
| CCT6A-R | 5’-AGGATGAAGGCCCATTTCGT-3’ |
| CCT6P1-F | 5’-TGCAGATGTCTTAACAGAGGC -3’ |
| CCT6P1-R | 5’-ATGAGTTTTTCTCTTTCTGCACTC-3’ |
| TXNIP-F | 5’-TGCAGATGTCTTAACAGAGGC -3’ |
| TXNIP-R | 5’-ATGAGTTTTTCTCTTTCTGCACTC-3’ |
| DDIT4-F | 5’-TGCAGATGTCTTAACAGAGGC -3’ |
| DDIT4-R | 5’-ATGAGTTTTTCTCTTTCTGCACTC-3’ |
| C-Myc-F | 5’-TGCAGATGTCTTAACAGAGGC -3’ |
| C-Myc-R | 5’-ATGAGTTTTTCTCTTTCTGCACTC-3’ |
| ALDOA-F | 5’-TCCATTGGCACCGAGAACACC-3’ |
| ALDOA-R | 5’-CTACCTTGATGCCCACAACACC-3’ |
| ENO1-F | 5’-TGAAGTCATCCTGCCAGTCCC-3’ |
| ENO1-R | 5’-CTACGTCCATGCCGATGACCA-3’ |
| HK2-F | 5’-GCTGTTTGACCACATTGCCGAA-3’ |
| HK2-R | 5’-ATTCACCACAGCCACAATGTCG-3’ |
| PGK1-F | 5’-TTCTAACAAGCTGACGCTGGA-3’ |
| PGK1-R | 5’-GCTCATAAGGACTACCGACT-3’ |
| PFKP-F | 5’-AGCTGCAGTGAAAACTACACC-3’ |
| PFKP-R | 5’-AGCACACAAATGGAATCATCGG-3’ |
| PKM-F | 5’-TCATTCATCCGCAAGGCATC-3’ |
| PKM-R | 5’-CAATGCCTAGATCACCACGAG-3’ |
| GPI-F | 5’-CAAGGACCGCTTCAACCACTT-3’ |
| GPI-R | 5’-CCAGGATGGGTGTGTTTGACC-3’ |
| TPI1-F | 5’-CCCGGCAGAAGCTAGATCCCAA-3’ |
| TPI1-R | 5’-ACAACCTTCTCAGTGATGCCAG-3’ |
| PDK1-F | 5’-CTTCTCAGGACACCATCCGTTC-3’ |
| PDK1-R | 5’-ATCATCTTGCAGGCCATACAGC-3’ |
| LDHA-F | 5’-CAGCCTTTTCCTTAGAACACC-3’ |
| LDHA-R | 5’-CACGTAGGTCAAGATATCCAC-3’ |
| PGM2-F | 5’-GTAGCAGCAGAAGGTTTGCC-3’ |
| PGM2-R | 5’-TGAAGGGCACAAAGGGGGTT-3’ |
| GLUT1-F | 5’-CTTTGTGGCCTTCTTTGAAGT-3’ |
| GLUT1-R | 5’-CCACACAGTTGCTCCACAT-3’ |
| HK1-F | 5’-CGCAGCTCCTGGCCTATTAC-3’ |
| HK1-R | 5’-GAGCCGCATGGCATAGAGAT-3’ |
| GLUT4-F | 5’-TGGAAGGAAAAGGGCCATGCTG-3’ |
| GLUT4-R | 5’-CAATGAGGAATCGTCCAAGGATG-3’ |
| G6P-F | 5’-GGTACACAGGCAAGACCATC-3’ |
| G6P-R | 5’-GTTTTGGCAATGTGAGTTCC-3’ |
| PFKL-F | 5’-CACAGGTGCCAACATCTTCCGCA-3’ |
| PFKL-R | 5’-TCATGTCGGTGCCGCAGAAGTCG-3’ |
| ALDOB-F | 5’-CACCATTCAAGGGCTTGATGGCCT-3’ |
| ALDOB-R | 5’-TTCCTGGATAGCGAGGCTGGAT-3’ |
| GAPDH-F | 5’-GCACCGTCAAGGCTGAGAAC-3’ |
| GAPDH-R | 5’-TGGTGAAGACGCCAGTGGA-3’ |
| PGAM1-F | 5’-ATGATGTCCCACCACCTCCGAT-3’ |
| PGAM1-R | 5’-ATCCTTCAGACTCTCACAGGAG-3’ |
| ENO2-F | 5’-TGAAGGCAGTGGACCACATCAACT-3’ |
| ENO2-R | 5’-AGAGACACACCCAGGATGGCATT-3’ |

**Supplementary Table S6.** The target sequences of siRNAs for target gene used in this study.

| CCT6P1-si1 | 5’- GGGACCATAAAGATCAAAT-3’ |
| --- | --- |
| CCT6P1-si2 | 5’- GCTGGAATCTTCTCTGAAA-3’ |
| CCT6A- si1 | 5’- GAGACATCAAACTTACTAA-3’ |
| CCT6A- si2  c-MYC-si1  c-MYC-si2 | 5’-GAAACTGATACAAGCTTAA-3’  5’-CCCAAGGUAGUUAUCCUUAAAdTdT-3’  5’-ACUGAAAGAUUUAGCCAUAAUdTdT-3’ |
